# Supplementary material for: Derivatives of the β-Crinane Amaryllidaceae Alkaloid Haemanthamine as Multi-Target Directed Ligands for Alzheimer’s Disease
Source: Molecules. 2019 Apr 3;24(7):1307. doi: 10.3390/molecules24071307 (PMC6480460; doi:10.3390/molecules24071307)
Supplement: Supplementary file 1 [file molecules-24-01307-s001.pdf]

Supplementary Material for:

Derivatives of the β-crinane Amaryllidaceae Alkaloid Haemanthamine as Multi-Target Directed Ligands for Alzheimer´s Disease

Eliška Kohelová [1], Rozálie Peřinová [1], Negar Maafi[1], Jan Korábečný [2,3], Daniela Hulcová [1,4], Jana Maříková [5], Tomáš Kučera [2], Loreto Martine-Gonzalez [6], Martina Hrabinova [2], Katarina Vorčáková [7], Lucie Nováková [8], Angela De Simone [9], Radim Havelek [10],Lucie Cahlíková [1]*

[1] ADINACO Research Group, Department of Pharmaceutical Botany, Faculty of Pharmacy, Charles University, Heyrovského 1203, 500 05 Hradec Králové, Czech Republic

[2] Department of Toxicoloxy and Military Pharmacy, Faculty of Military Health Sciences, University of Defence, Třebešská 1575, 500 05 Hradec Králové, Czech Republic

[3] Department Biomedical Research Centre, University Hospital Hradec Kralove, Sokolska 581, 500 05 Hradec Králové, Czech Republic

[4] Department of Pharmacognosy, Faculty of Pharmacy, Charles University, Heyrovského 1203, 500 05 Hradec Králové, Czech Republic

[5] Department of Organic and Bioorganic Chemistry, Faculty of Pharmacy, Charles University, Heyrovského 1203, 500 05 Hradec Králové, Czech Republic

[6] Centro de Investigaciones Biológicas-CSIC, Avenida Ramiro de Maeztu 9, 28040 Madrid, Spain

[7] Deaprtment of Biological and Biochemical Sciences, Faculty of Chemical technology, University of Pardubice, Studentská 95, 532 10 Pardubice, Czech Republic

[8] Department of Analytical Chemistry, Faculty of Pharmacy, Charles University, Heyrovského 1203, 500 05 Hradec Králové, Czech Republic

[9] Department for Life Quality Studies, University of Bologna, Corso D'Augusto 237, 47921 Rimini, Italy

[10] Department of Medicinal Biochemistry, Faculty of Medicine, Charles University, Zborovská 2089, 500 03 Hradec Králové, Czech Republic

* Correspondence: cahlikova@faf.cuni.cz; Tel.: +420-495067311; Fax: +420-495067162

Analytical spectra of haemanthamine:

HPLC (UV/VIS)

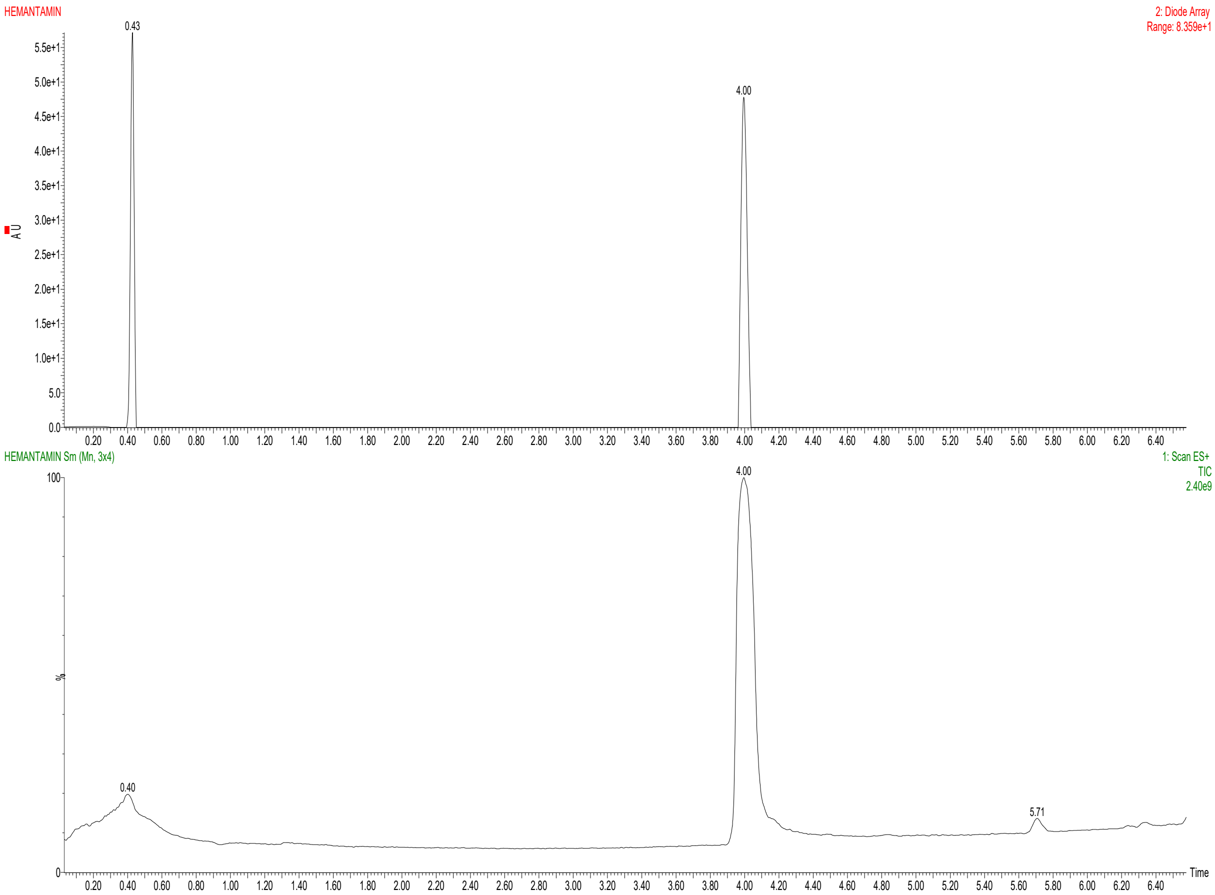

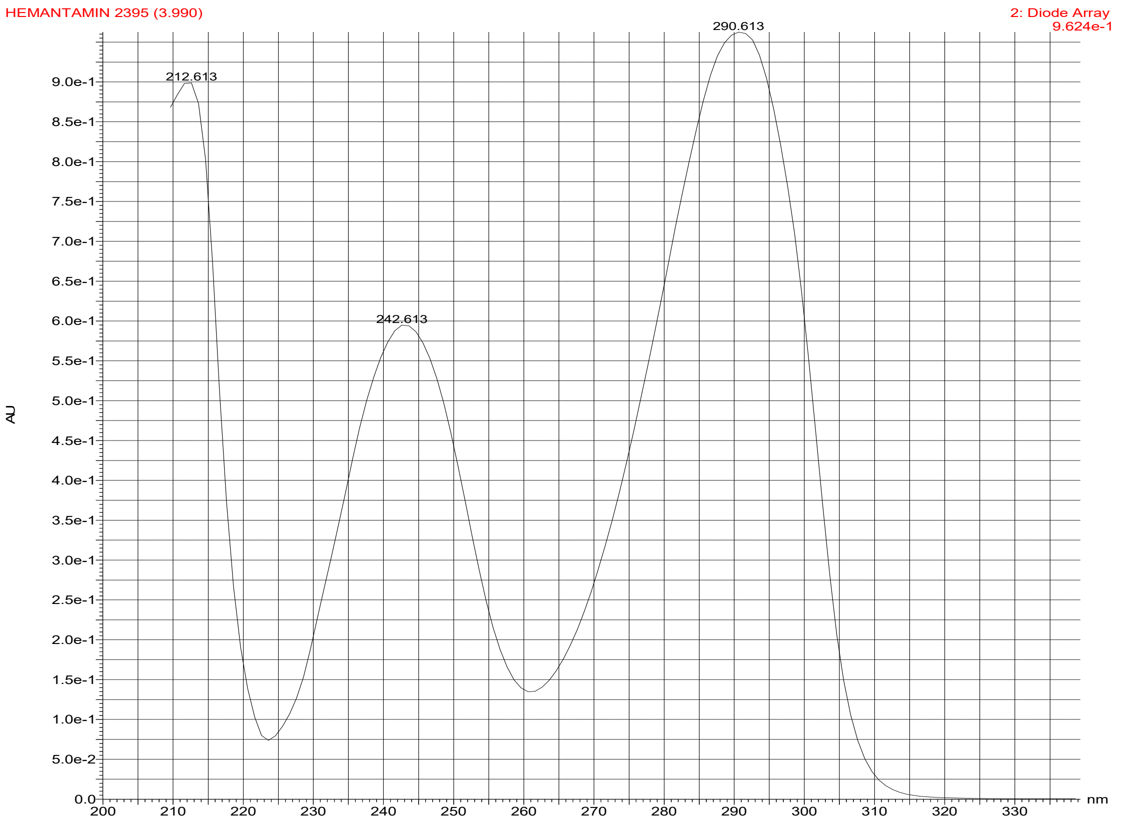
HEMANTAMIN 2395 (3.990)
2: Diode Array
9.624e-1
290.613
212.613
242.613
AU
9.0e-1
8.5e-1
8.0e-1
7.5e-1
7.0e-1
6.5e-1
6.0e-1
5.5e-1
5.0e-1
4.5e-1
4.0e-1
3.5e-1
3.0e-1
2.5e-1
2.0e-1
1.5e-1
1.0e-1
5.0e-2
0.0
200 210 220 230 240 250 260 270 280 290 300 310 320 330
nm

HPLC/MS

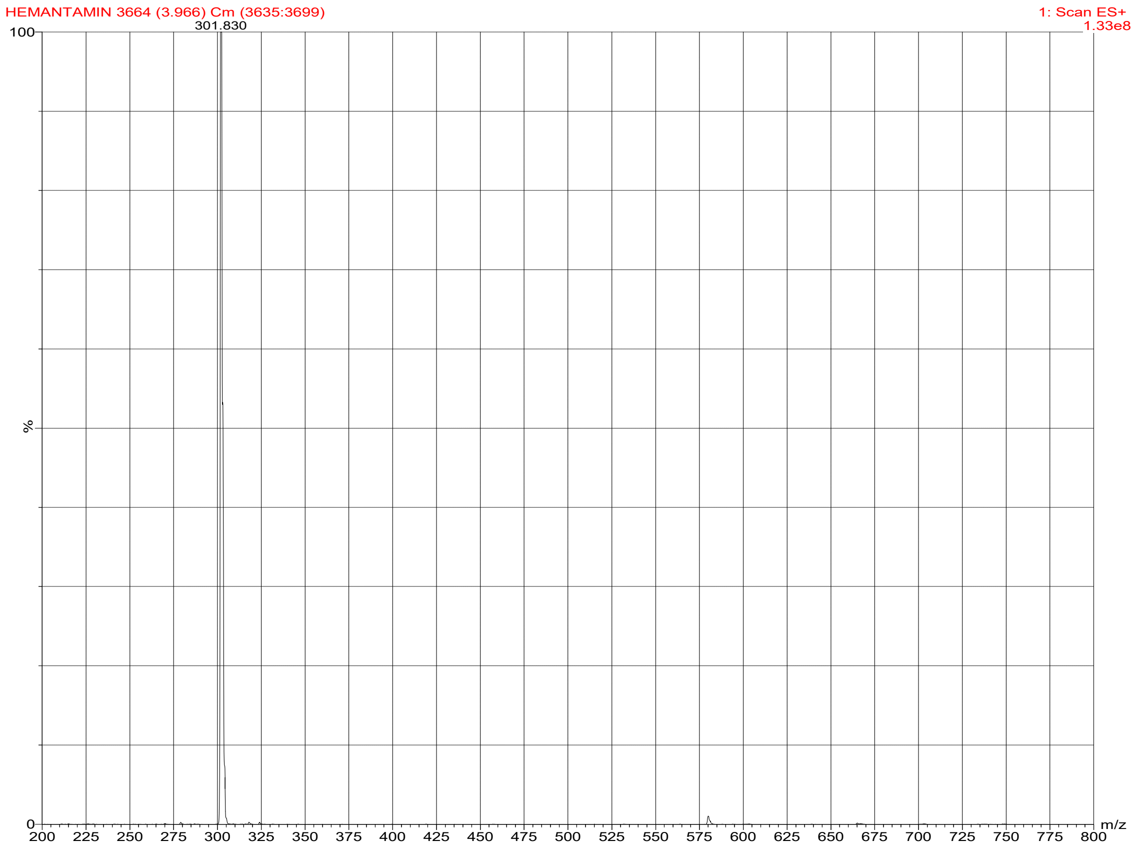
HEMANTAMIN 3664 (3.966) Cm (3635:3699)
1: Scan ES+
1.33e8
301.830
100
%
0
200 225 250 275 300 325 350 375 400 425 450 475 500 525 550 575 600 625 650 675 700 725 750 775 800
m/z

^{1}H-NMR

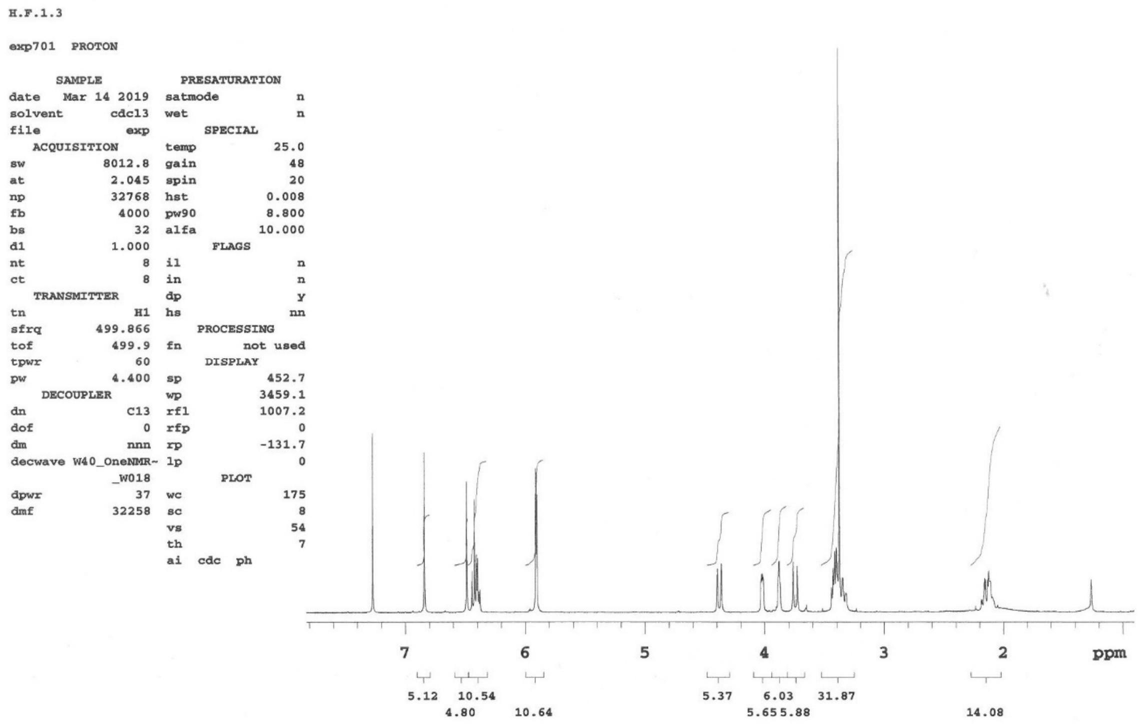

^{13}C-NMR

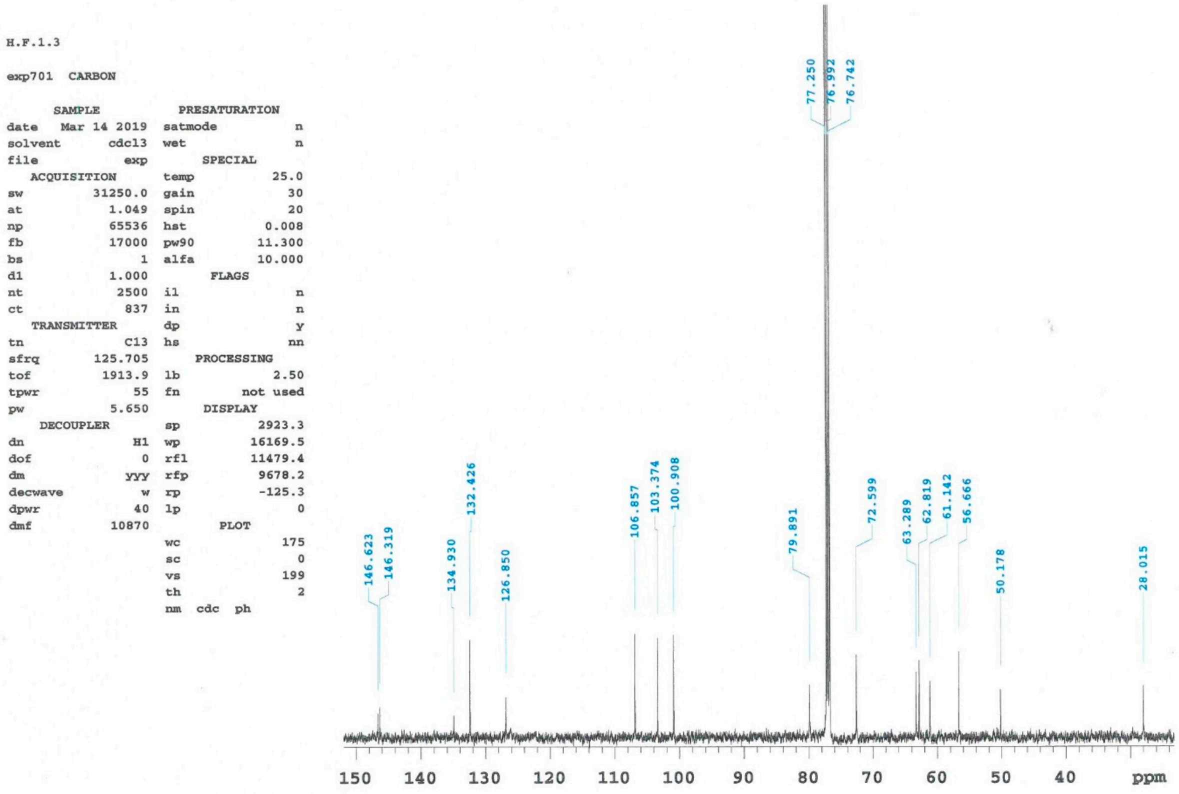

Analytical spectra of prepared derivatives:

11-*O*-Propionylhaemanthamine (1b)

ESI-HRMS

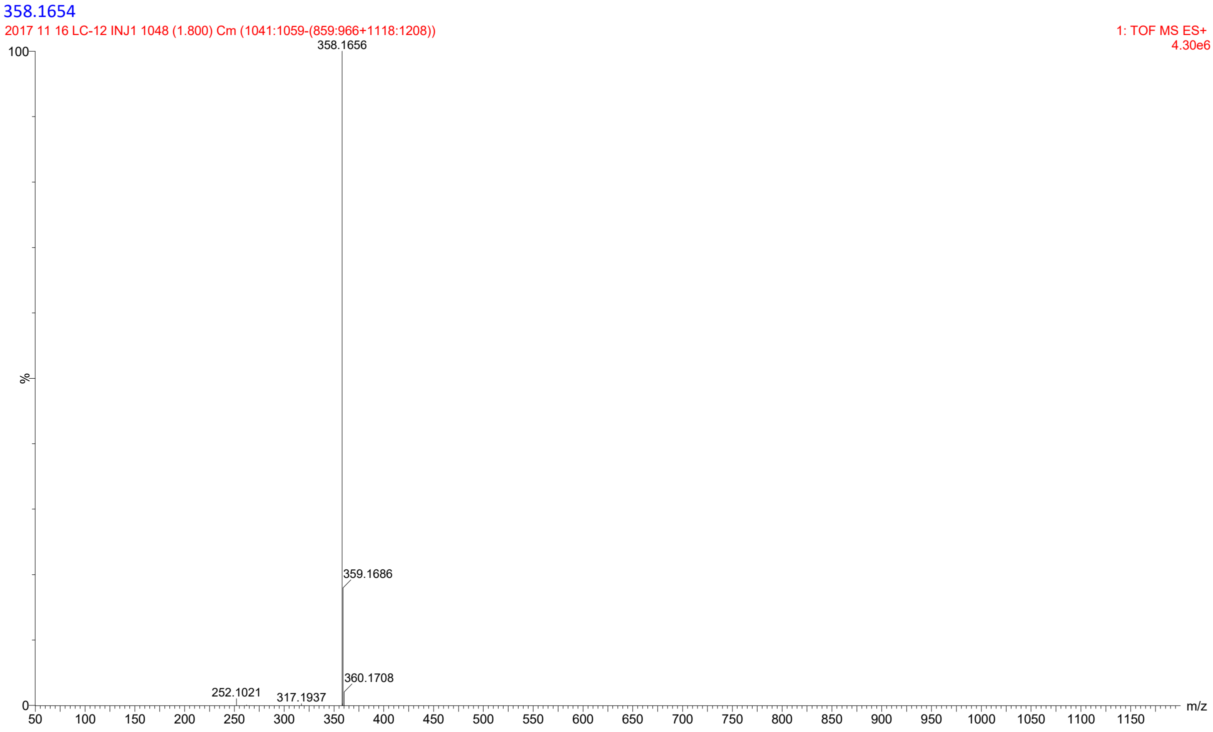

EI-MS

File :C:\msdchem\1\data\SNAP\2148LC.D
Operator :
Acquired : 21 Apr 2015 15:55 using AcqMethod LUCKA_7.M
Instrument : GCMS
Sample Name: LC-12
Misc Info :
Vial Number: 14

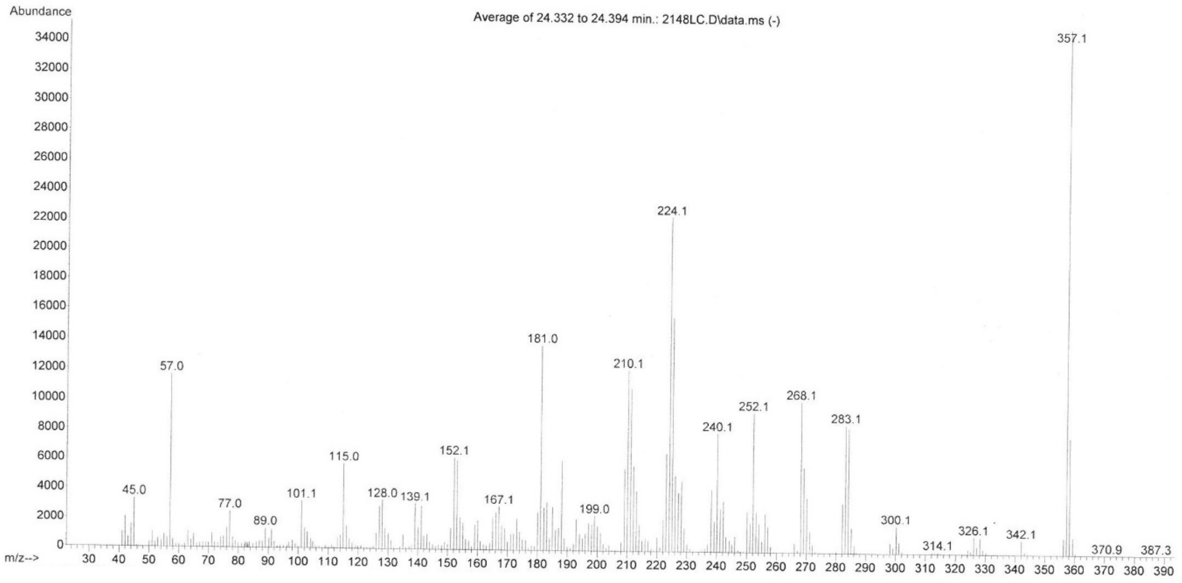

^{1}H-NMR

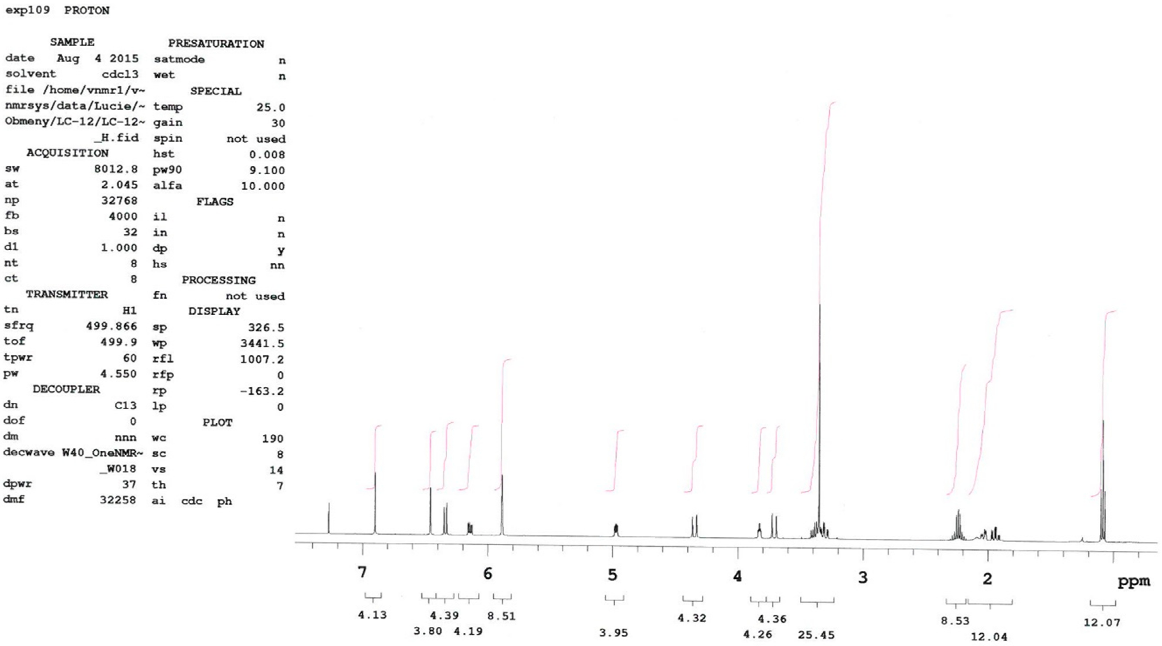

^{13}C-NMR

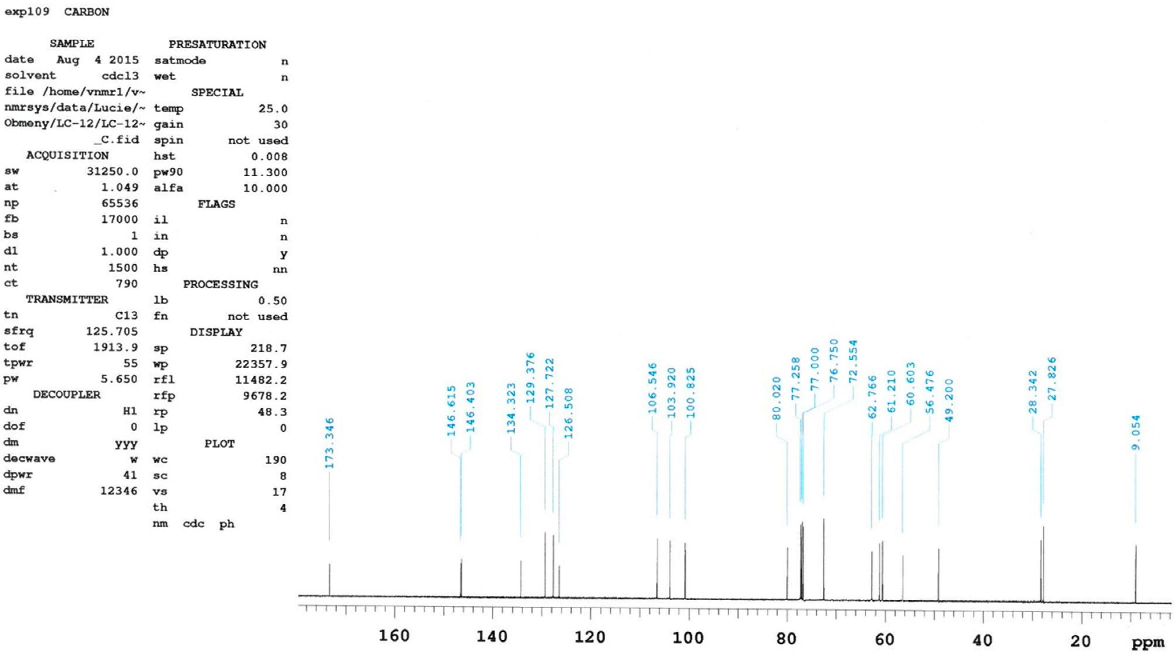

11-*O*-Pentanoylhaemanthamine (1d)

ESI-HRMS

386.1967

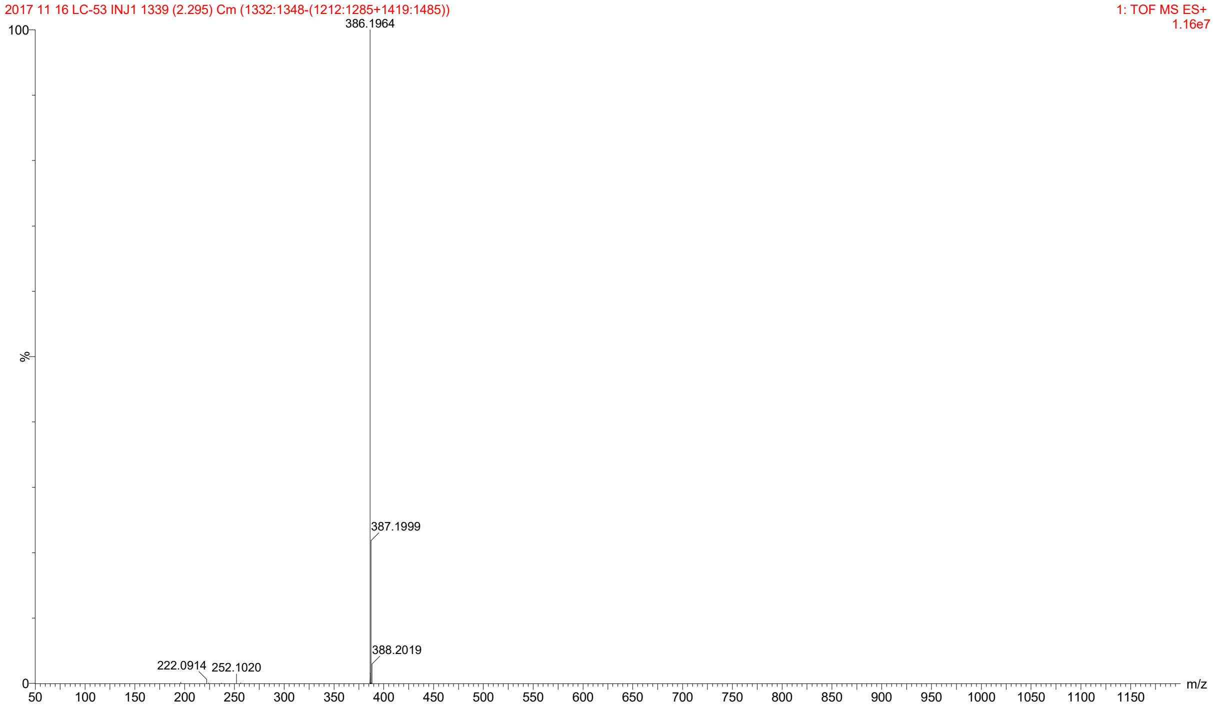

EI-MS

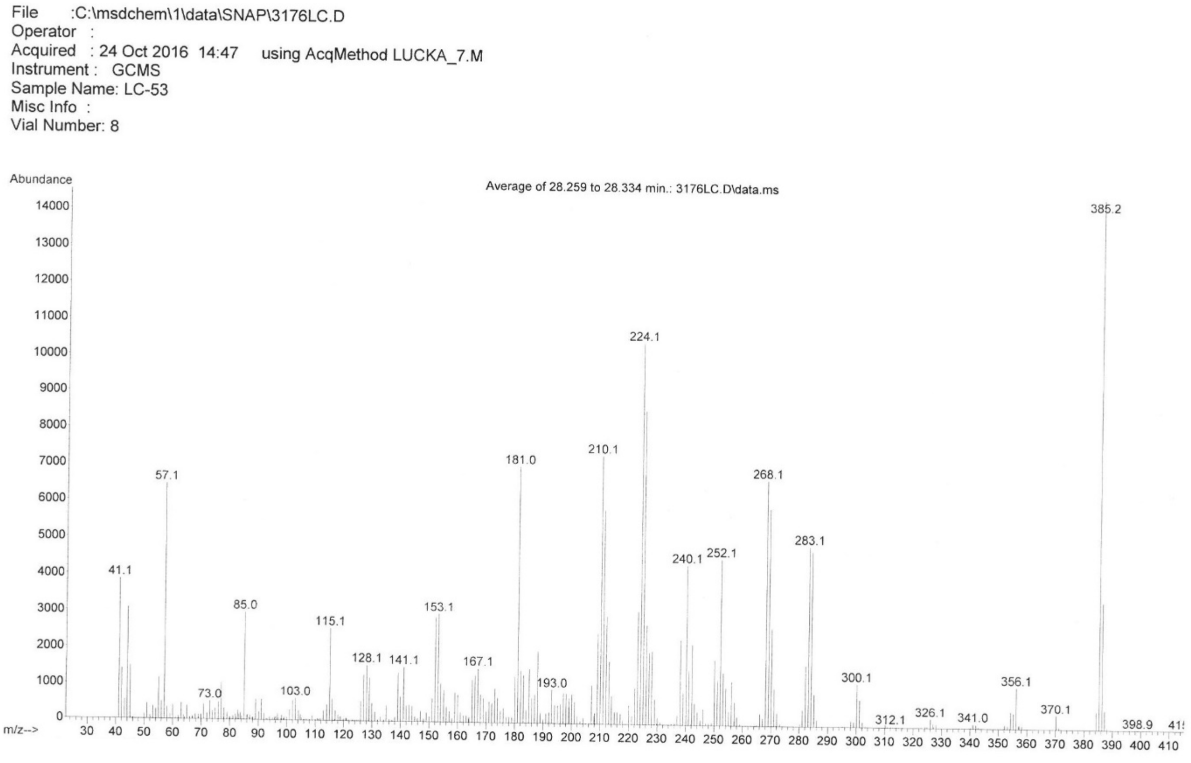

^{1}H-NMR

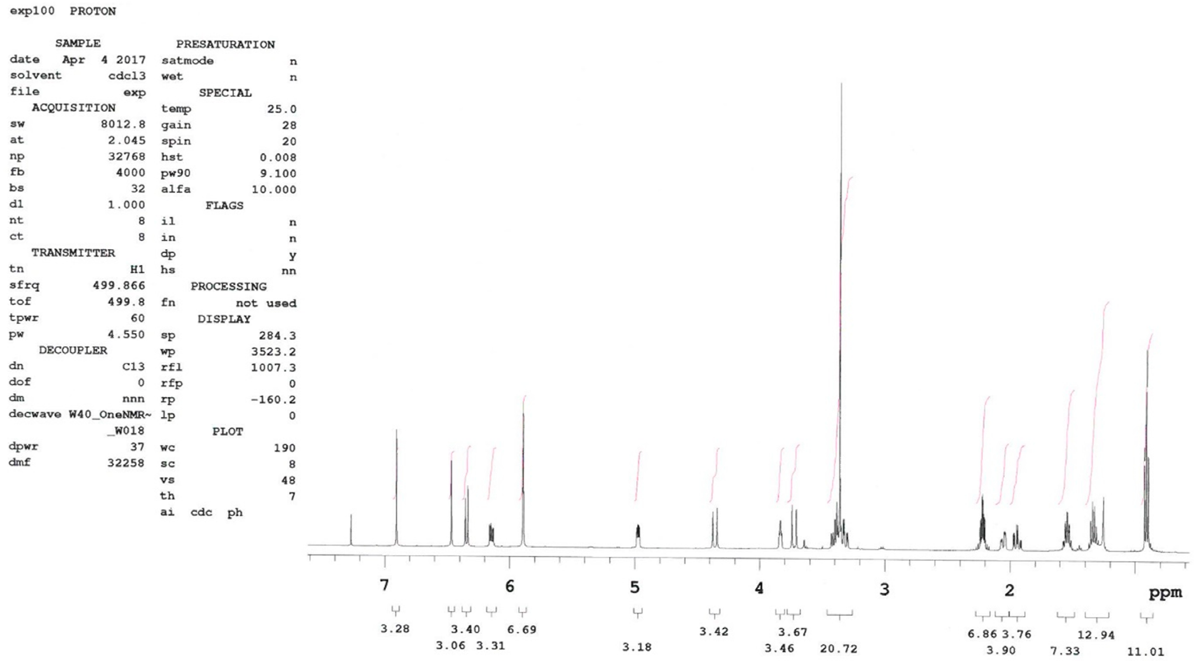

^{13}C-NMR

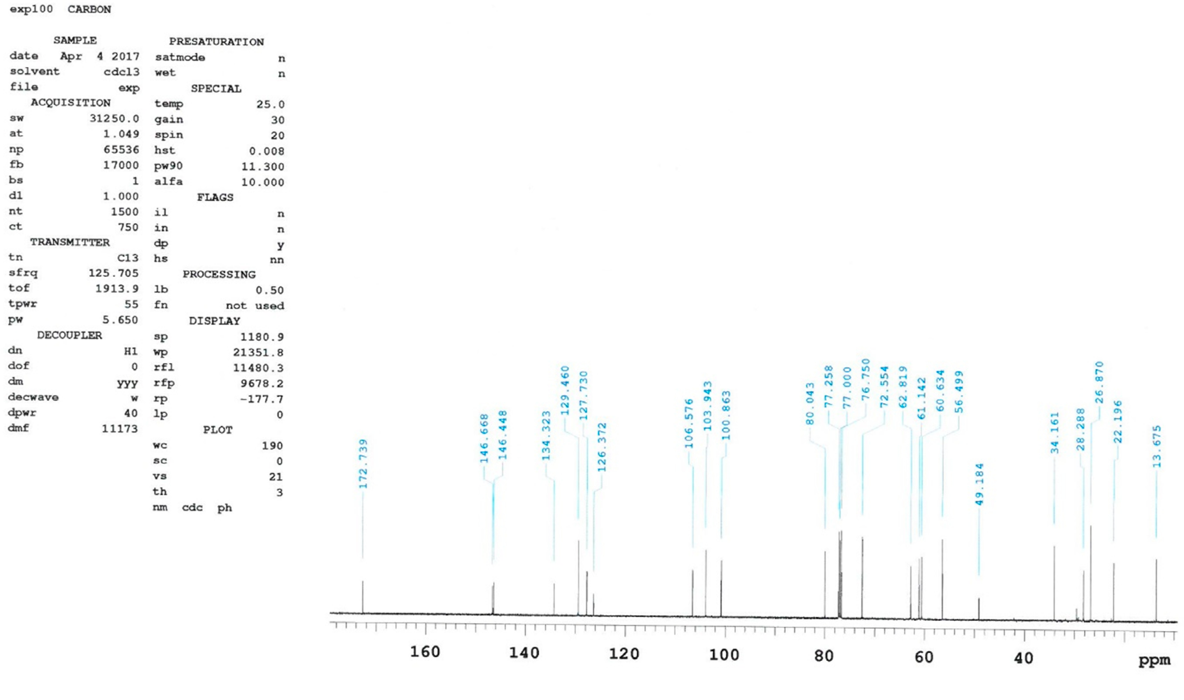

11-O-Hexanoylhaemanthamine (1e)

ESI-HRMS

400.2124

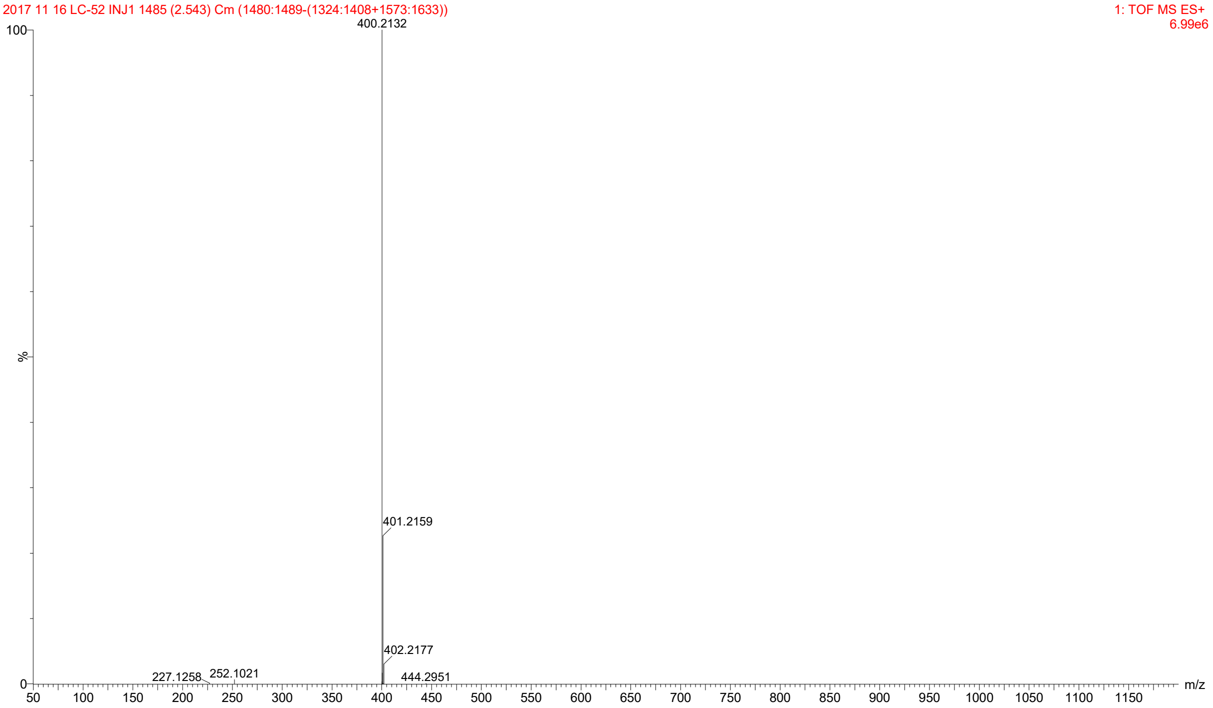

EI-MS

File :C:\msdchem\1\data\SNAP\3172LC.D
Operator :
Acquired : 18 Oct 2016 13:31 using AcqMethod LUCKA_7.M
Instrument : GCMS
Sample Name: LC-52
Misc Info :
Vial Number: 5

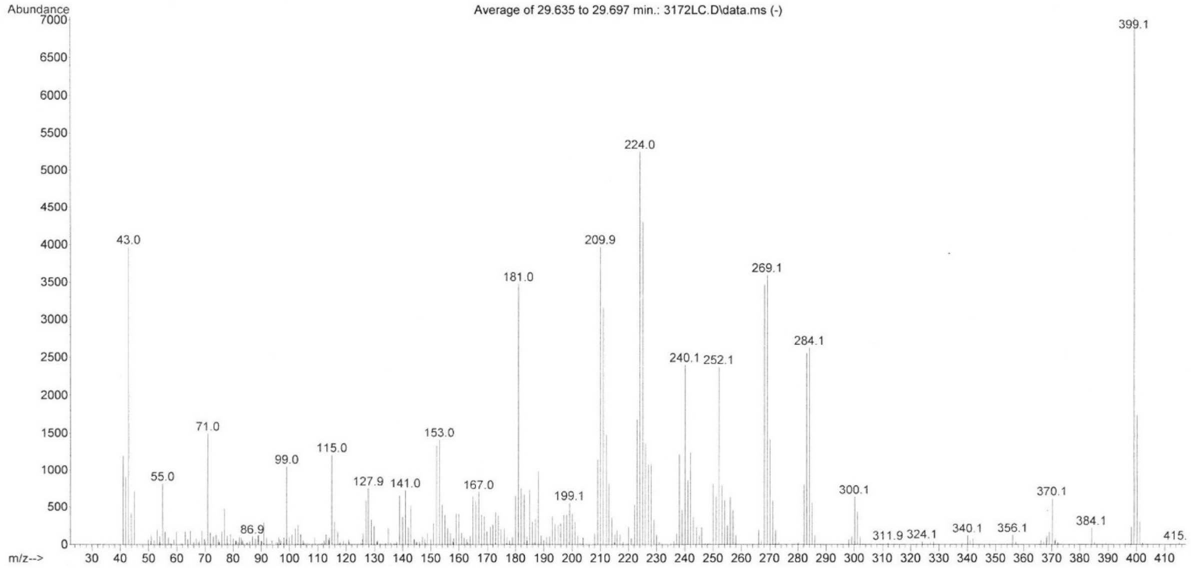

^{1}H-NMR

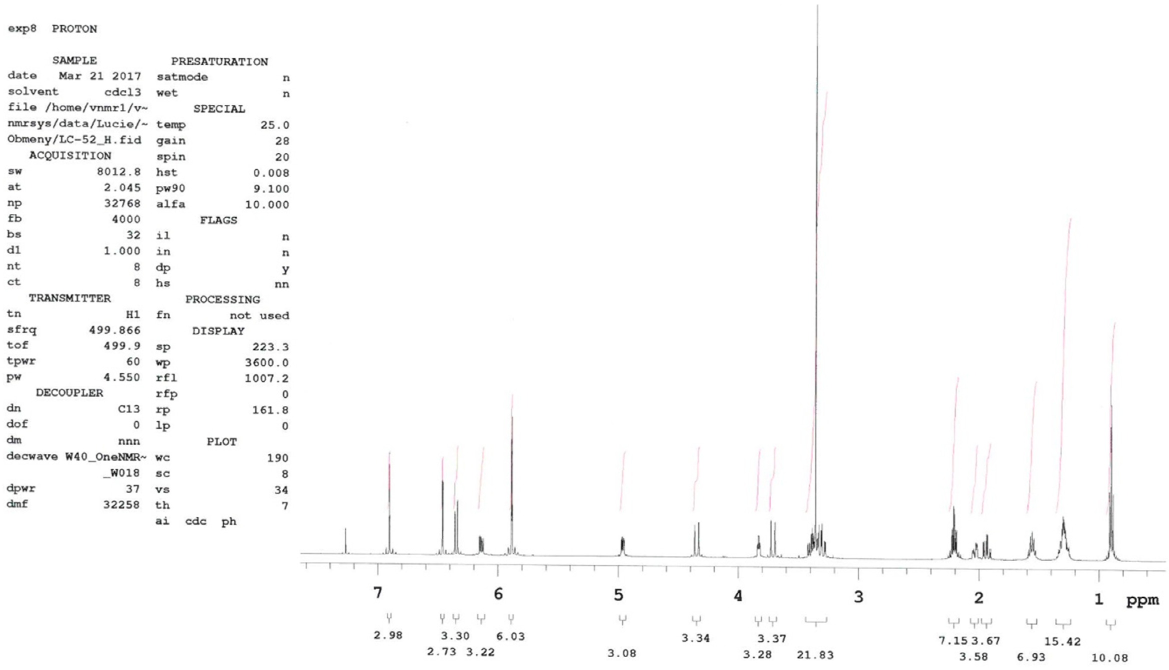

^{13}C-NMR

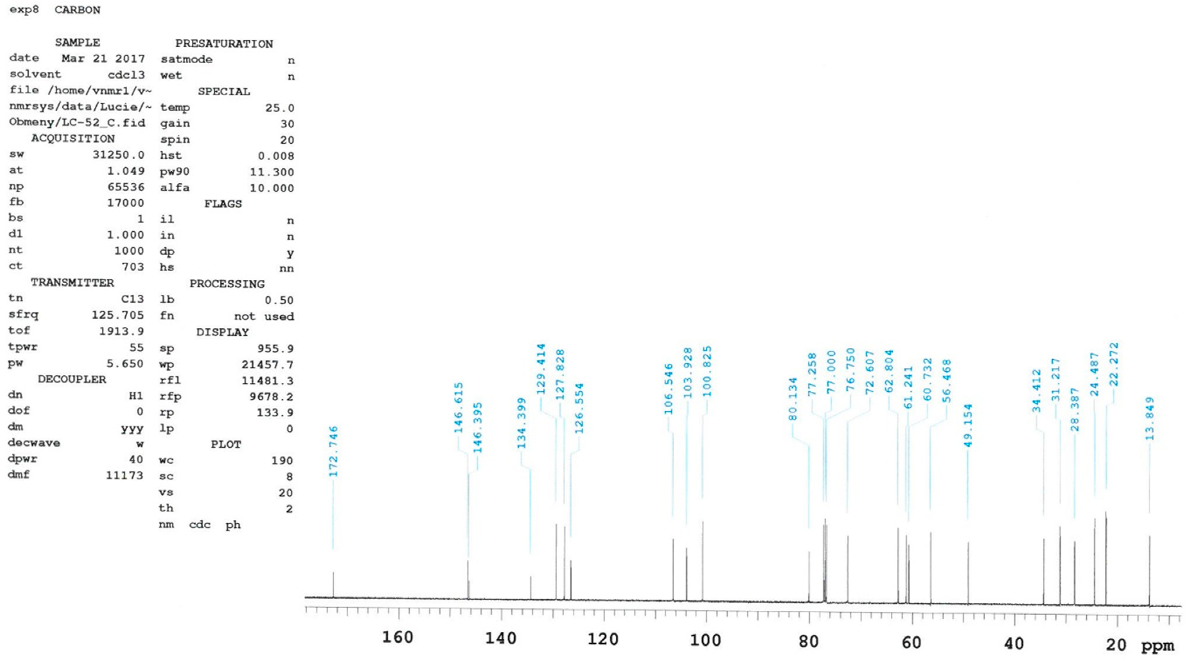

11-O-Butanoylhaemanthamine (1f)

EI-MS

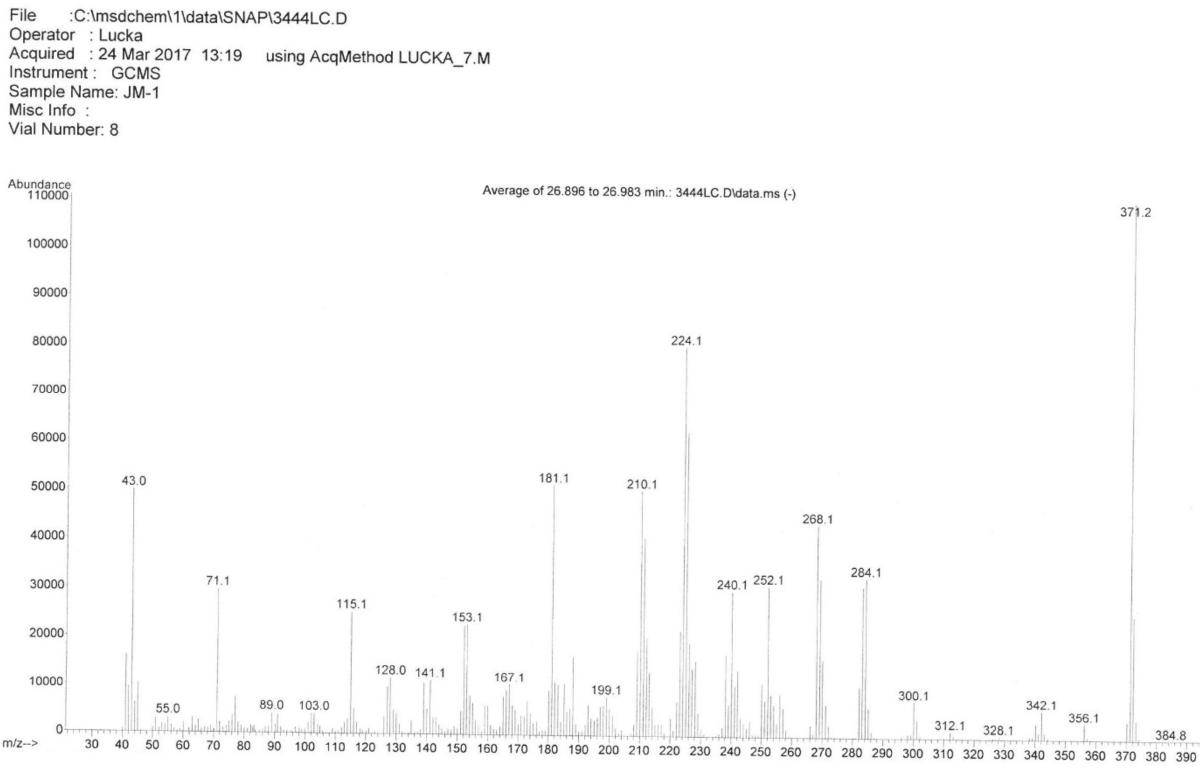

^{1}H-NMR

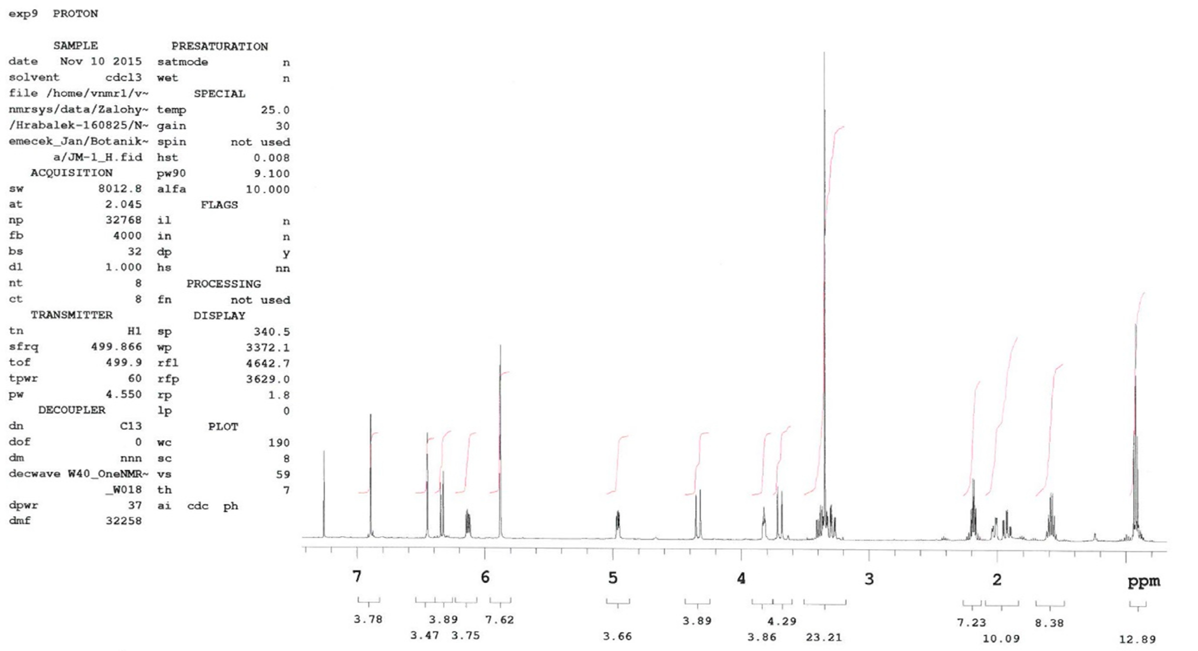

^{13}C-NMR

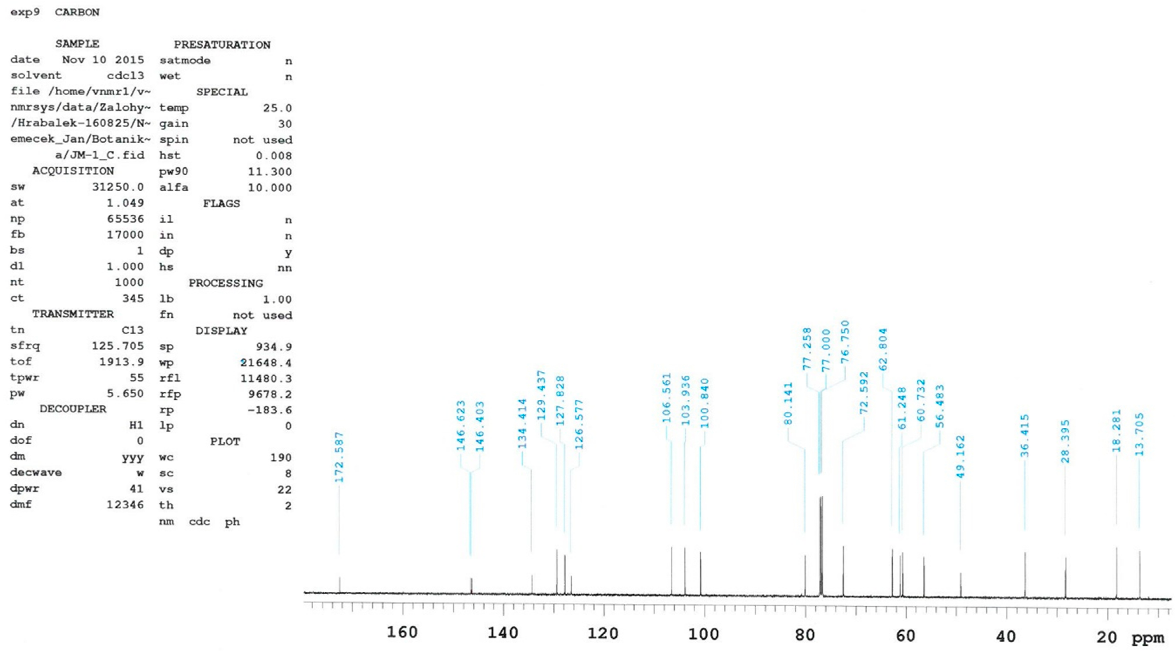

11-O-Benzoylhaemanthamine (1g)

ESI-HRMS

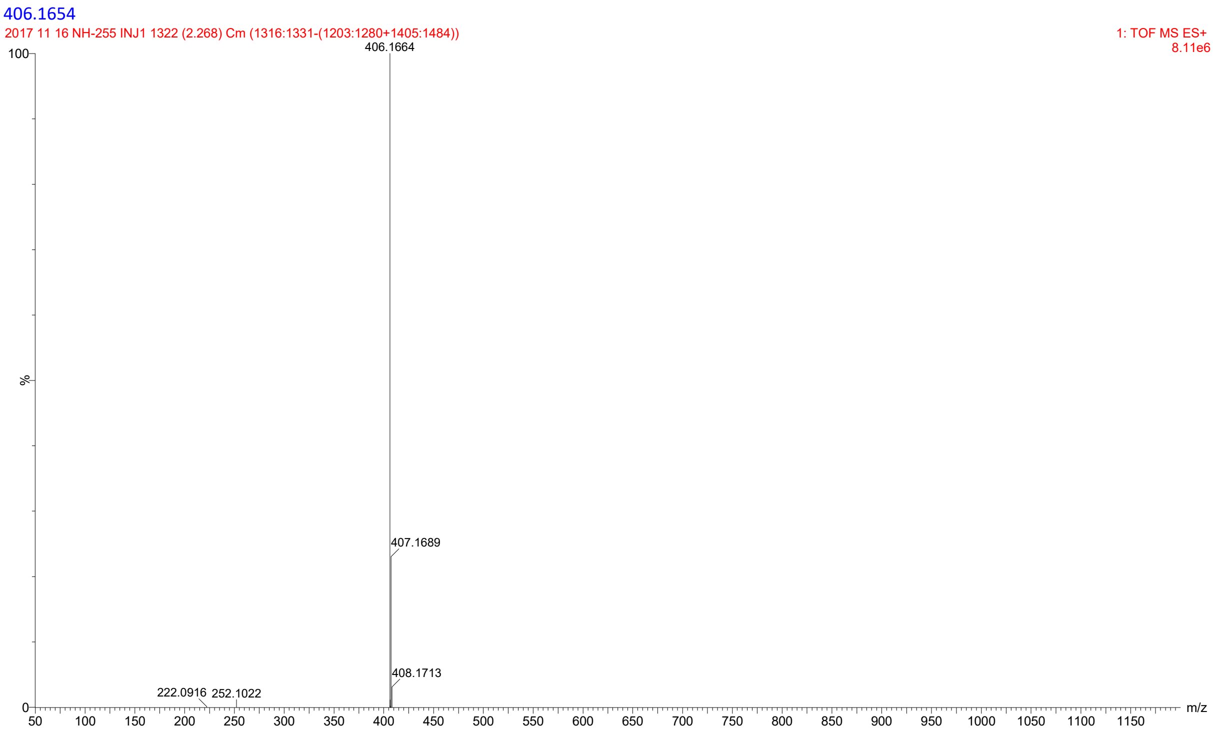

EI-MS

File :C:\msdchem\1\data\SNAP\3828LC.D
Operator : Lucka
Acquired : 17 Jan 2018 10:14 using AcqMethod LUCKA_7.M
Instrument : GCMS
Sample Name: LC-118
Misc Info :
Vial Number: 13

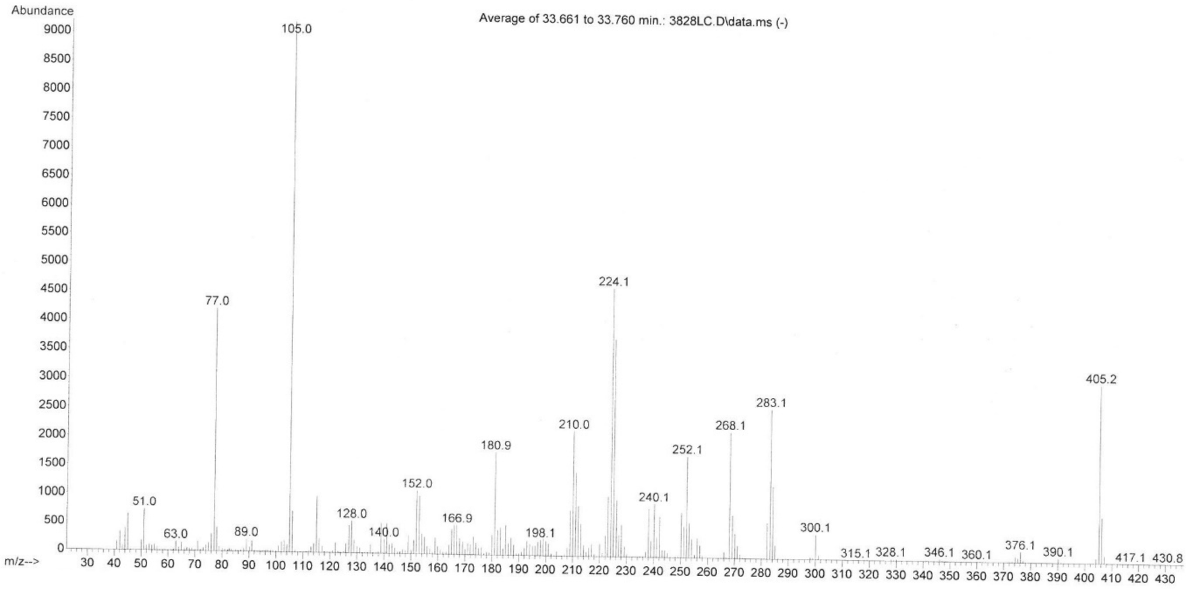

¹H-NMR

```
exp39  PROTON

      SAMPLE              PRESATURATION
date    Jan 23 2018   satmode            n
solvent       cdcl3   wet                n
file /home/vnmr1/v~         SPECIAL
nmrsys/data/Lucie/~   temp            25.0
Obmeny/LC-118_Ha.f~   gain              24
                 id   spin              20
   ACQUISITION        hst            0.008
sw           8012.8   pw90           8.700
at            2.045   alfa          10.000
np            32768          FLAGS
fb             4000   il                 n
bs               32   in                 n
d1            1.000   dp                 y
nt                8   hs                nn
ct                8        PROCESSING
   TRANSMITTER        fn          not used
tn               H1          DISPLAY
sfrq        499.866   sp             507.9
tof           499.9   wp            3689.0
tpwr             60   rfl           1007.2
pw            4.350   rfp                0
    DECOUPLER         rp             -97.1
dn              C13   lp                 0
dof               0          PLOT
dm              nnn   wc               175
decwave W40_OneNMR~   sc                 8
              _W018   vs                43
dpwr             37   th                 7
dmf           32258   ai   cdc  ph
```

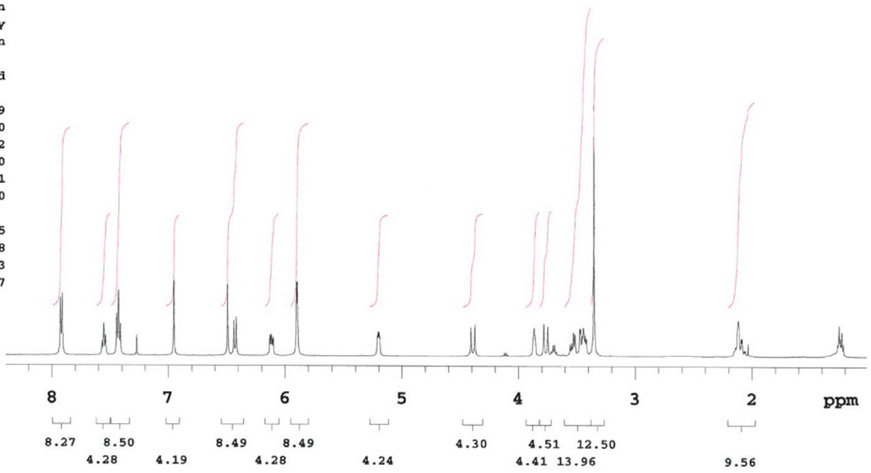

^{13}C-NMR

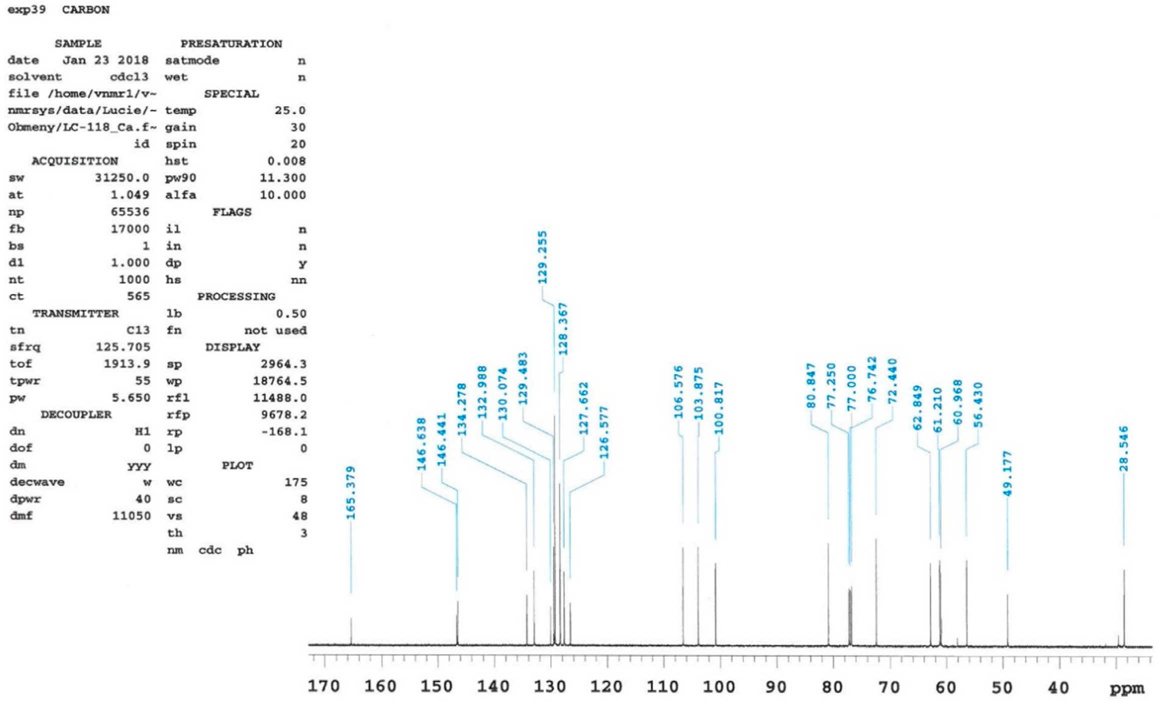

11-O-(3-chlorobenzoyl)haemanthamine (1h)

ESI-HRMS

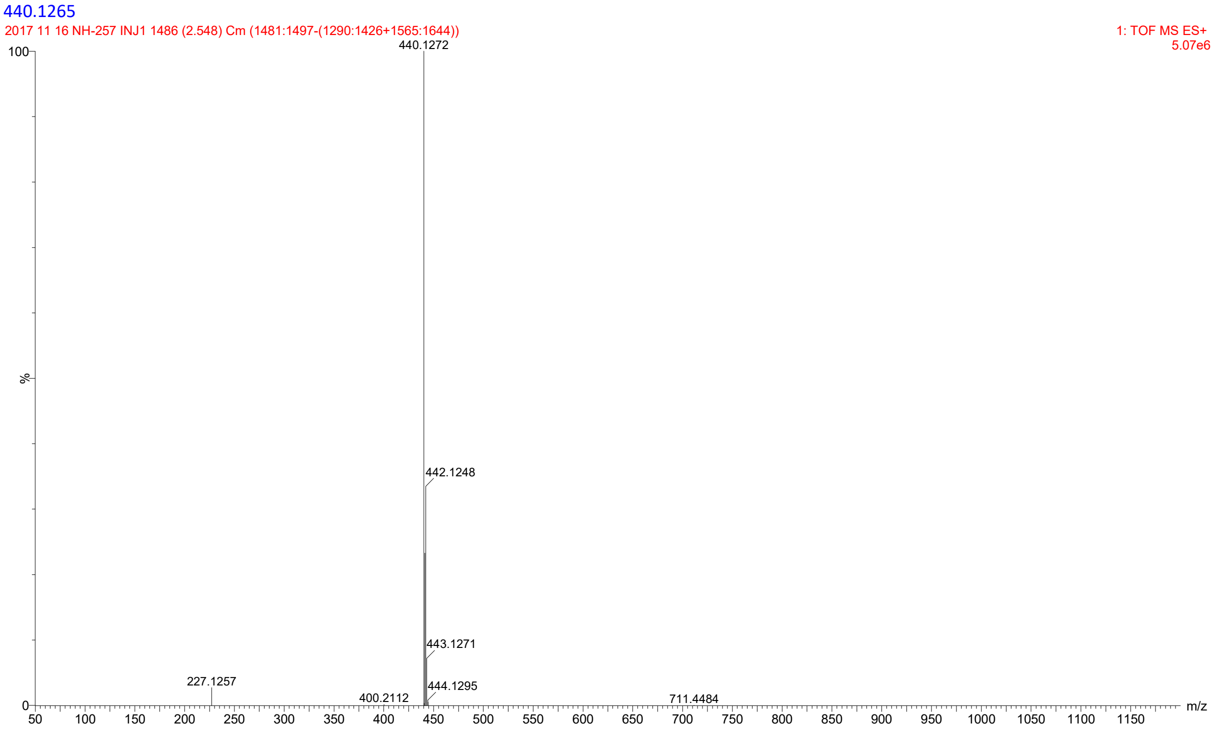

EI-MS

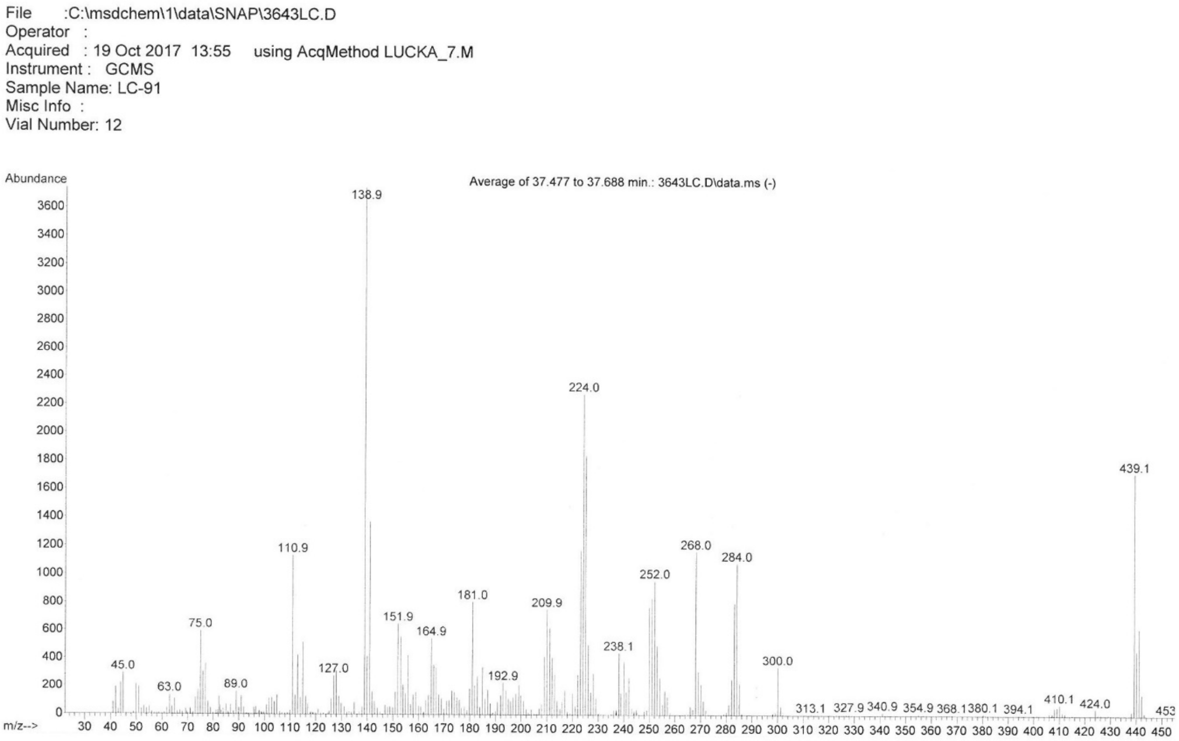
File :C:\msdchem\1\data\SNAP\3643LC.D
Operator :
Acquired : 19 Oct 2017 13:55 using AcqMethod LUCKA_7.M
Instrument : GCMS
Sample Name: LC-91
Misc Info :
Vial Number: 12
Abundance
Average of 37.477 to 37.688 min.: 3643LC.D\data.ms (-)
m/z-->
138.9
224.0
439.1
110.9
268.0
284.0
252.0
181.0
209.9
151.9
164.9
75.0
238.1
300.0
45.0
127.0
192.9
63.0
89.0
410.1
424.0
453

^{1}H-NMR

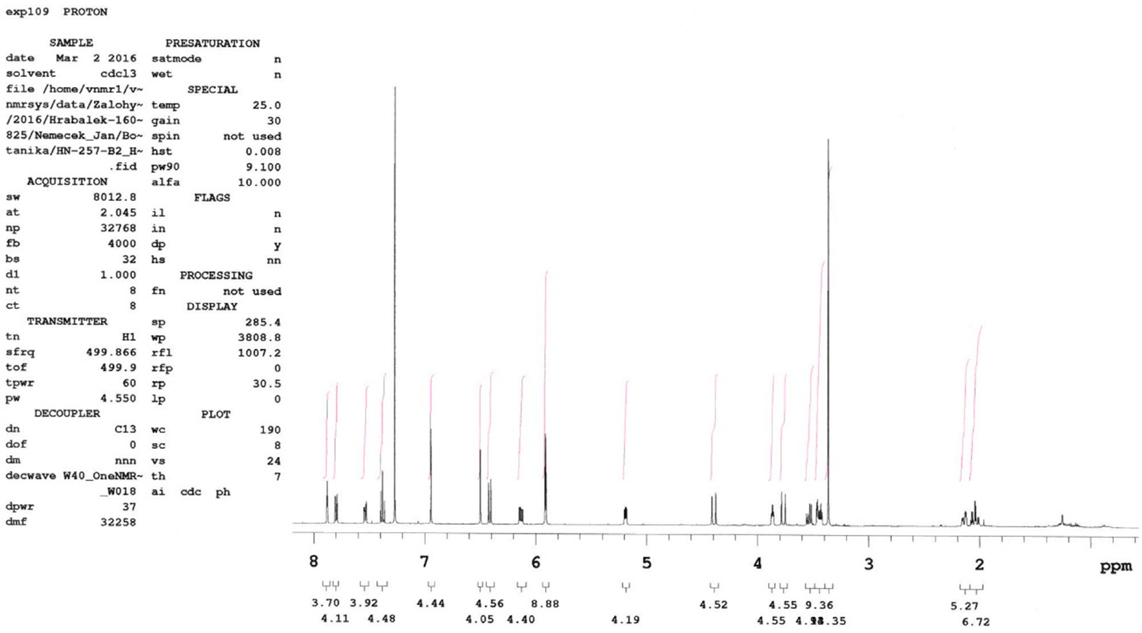
exp109 PROTON
SAMPLE
date Mar 2 2016
solvent cdcl3
file /home/vnmr1/v~
nmrsys/data/Zalohy~
/2016/Hrabalek-160~
825/Nemecek_Jan/Bo~
tanika/HN-257-B2_H~
.fid
ACQUISITION
sw 8012.8
at 2.045
np 32768
fb 4000
bs 32
d1 1.000
nt 8
ct 8
TRANSMITTER
tn H1
sfrq 499.866
tof 499.9
tpwr 60
pw 4.550
DECOUPLER
dn C13
dof 0
dm nnn
decwave W40_OneNMR~
_W018
dpwr 37
dmf 32258
PRESATURATION
satmode n
wet n
SPECIAL
temp 25.0
gain 30
spin not used
hst 0.008
pw90 9.100
alfa 10.000
FLAGS
il n
in n
dp y
hs nn
PROCESSING
fn not used
DISPLAY
sp 285.4
wp 3808.8
rfl 1007.2
rfp 0
rp 30.5
lp 0
PLOT
wc 190
sc 8
vs 24
th 7
ai cdc ph
ppm
3.70 3.92 4.44 4.56 8.88 4.52 4.55 9.36 5.27
4.11 4.48 4.05 4.40 4.19 4.55 6.72

^{13}C-NMR

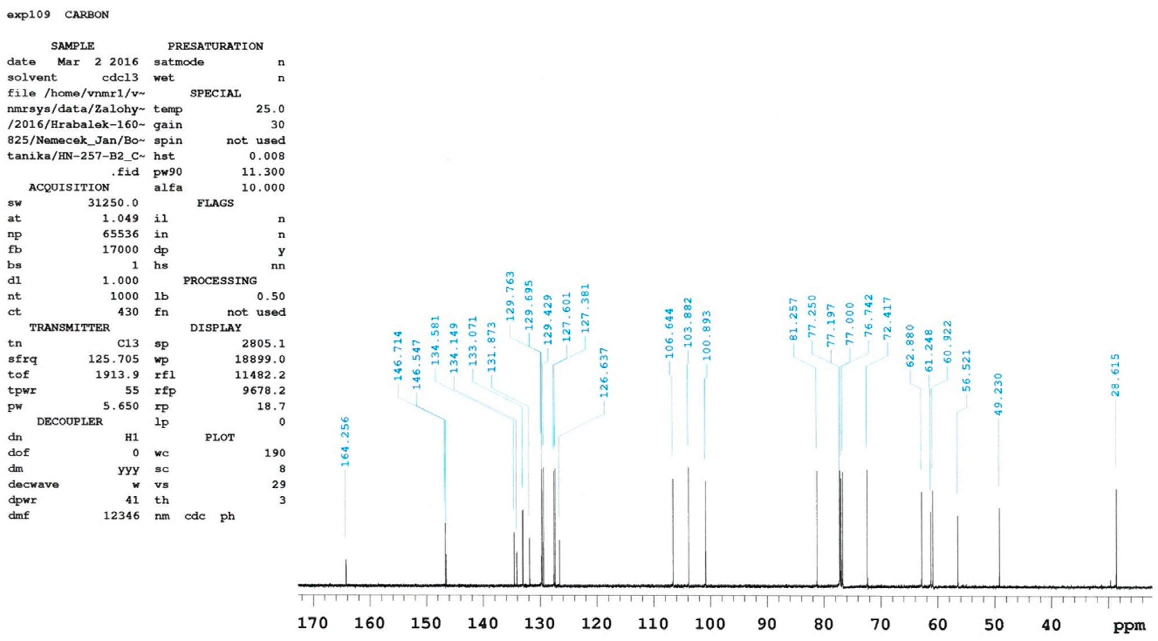

11-O-(3-bromobenzoyl)haemanthamine (1i)

ESI-HRMS

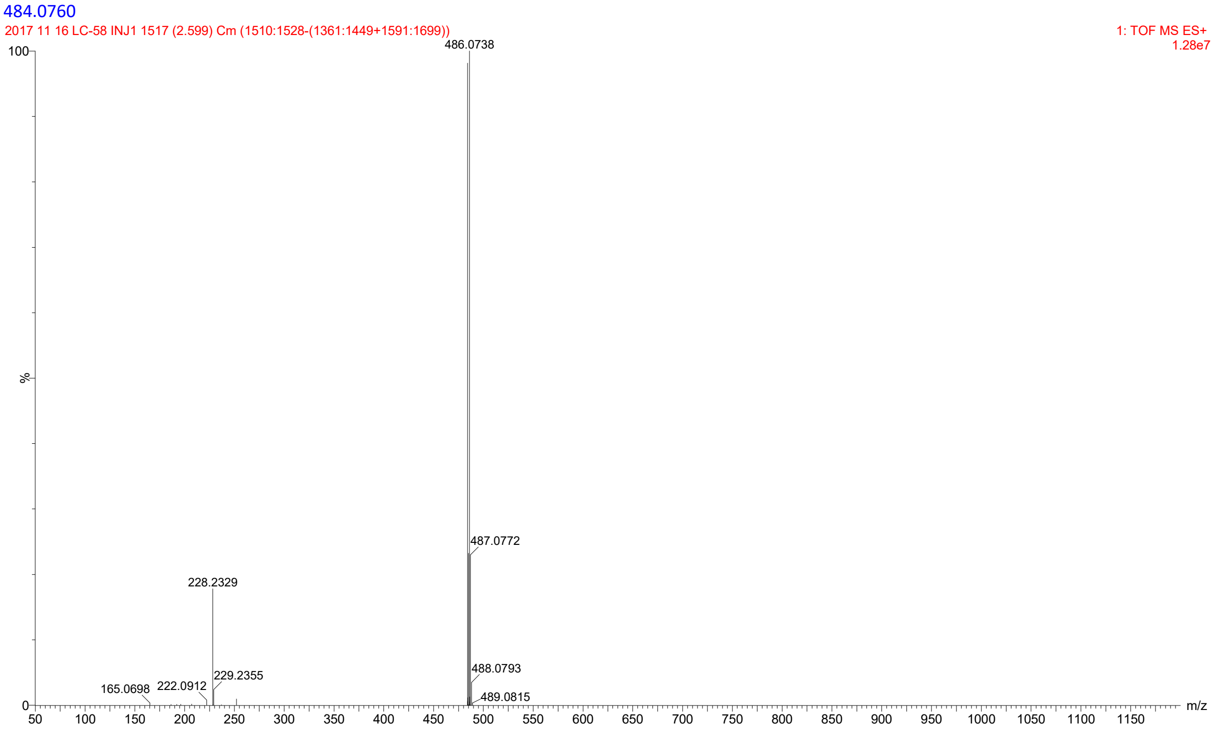

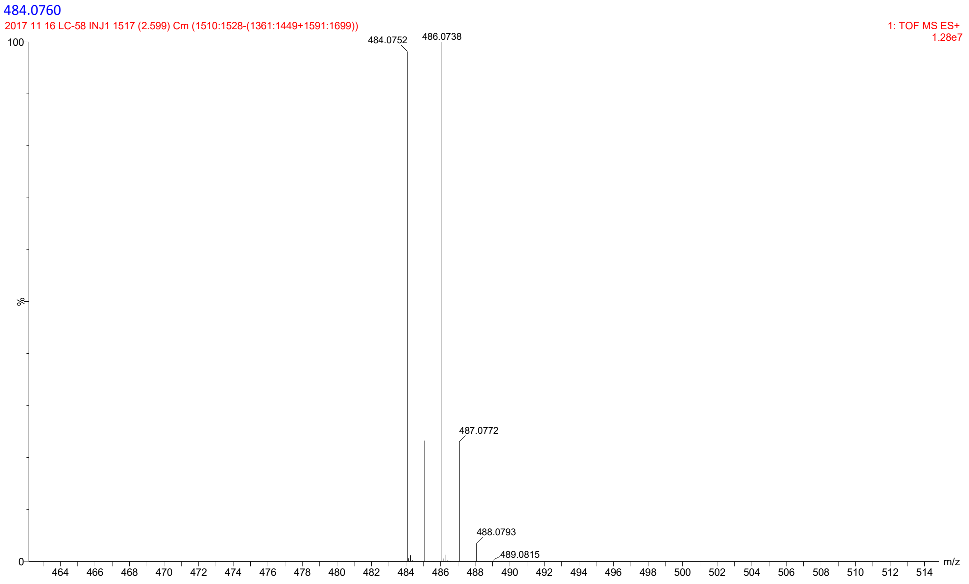

484.0760
2017 11 16 LC-58 INJ1 1517 (2.599) Cm (1510:1528-(1361:1449+1591:1699))
1: TOF MS ES+
1.28e7
484.0752
486.0738
487.0772
488.0793
489.0815
%
m/z

EI-MS

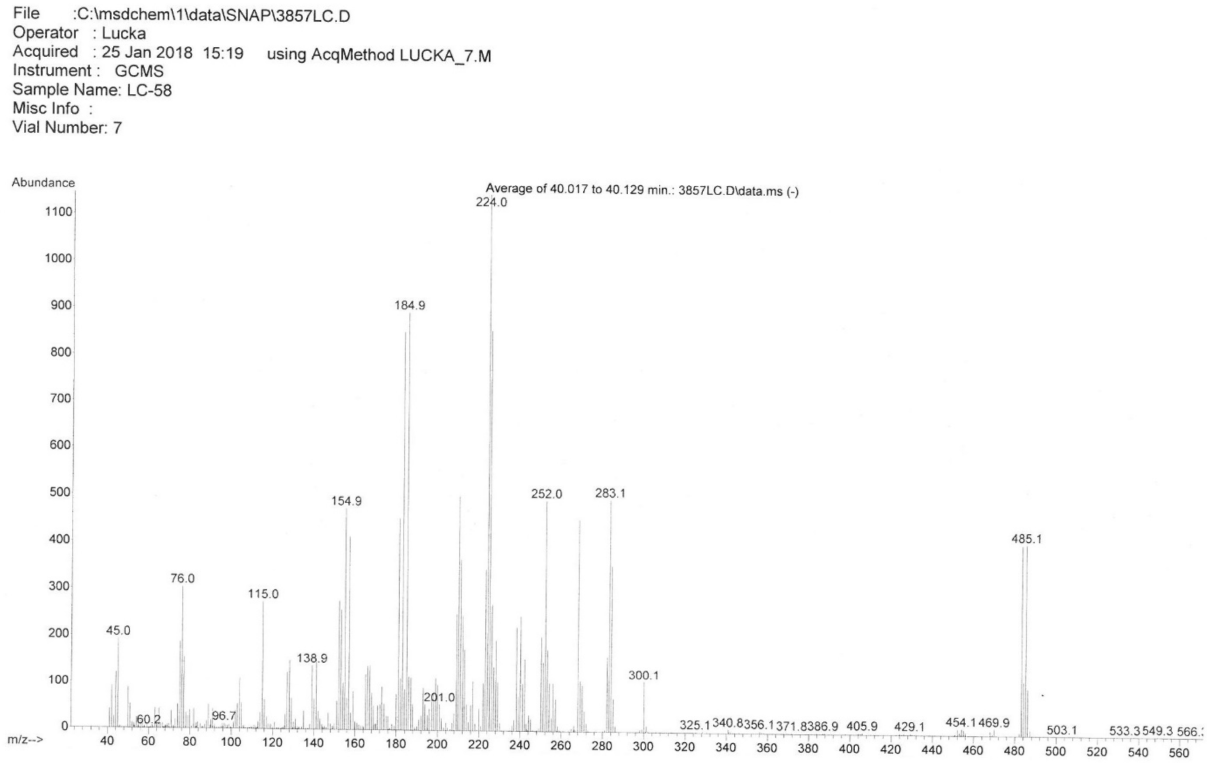

File :C:\msdchem\1\data\SNAP\3857LC.D
Operator : Lucka
Acquired : 25 Jan 2018 15:19 using AcqMethod LUCKA_7.M
Instrument : GCMS
Sample Name: LC-58
Misc Info :
Vial Number: 7
Abundance
Average of 40.017 to 40.129 min.: 3857LC.D\data.ms (-)
224.0
184.9
154.9
252.0
283.1
485.1
76.0
115.0
45.0
138.9
300.1
201.0
96.7
60.2
m/z-->

^{1}H-NMR

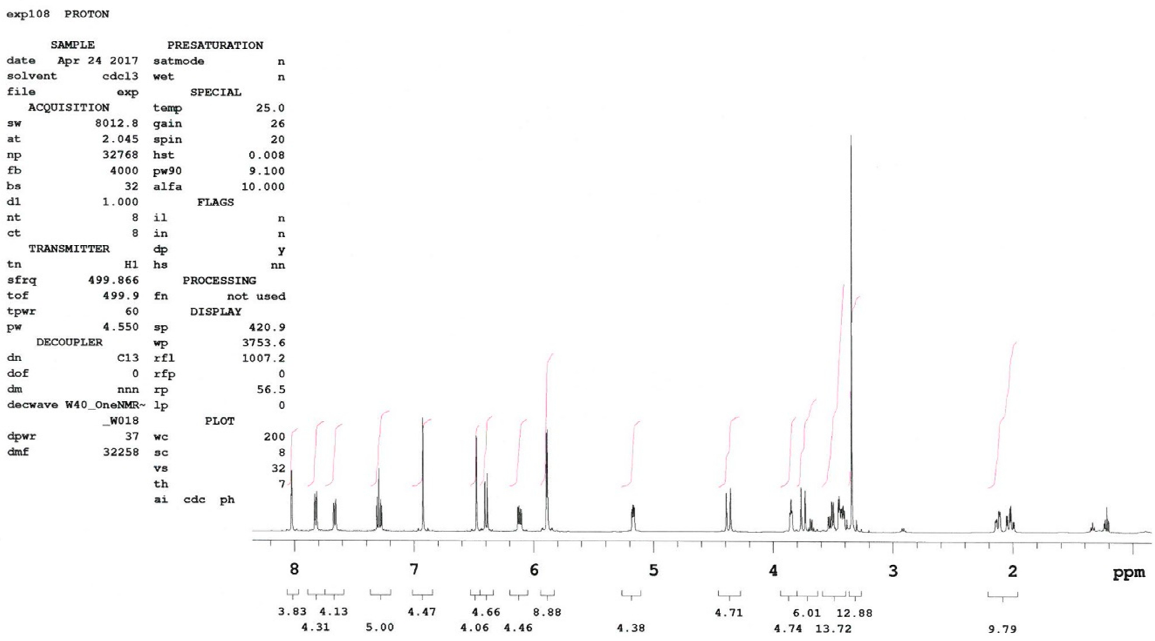

^{13}C-NMR

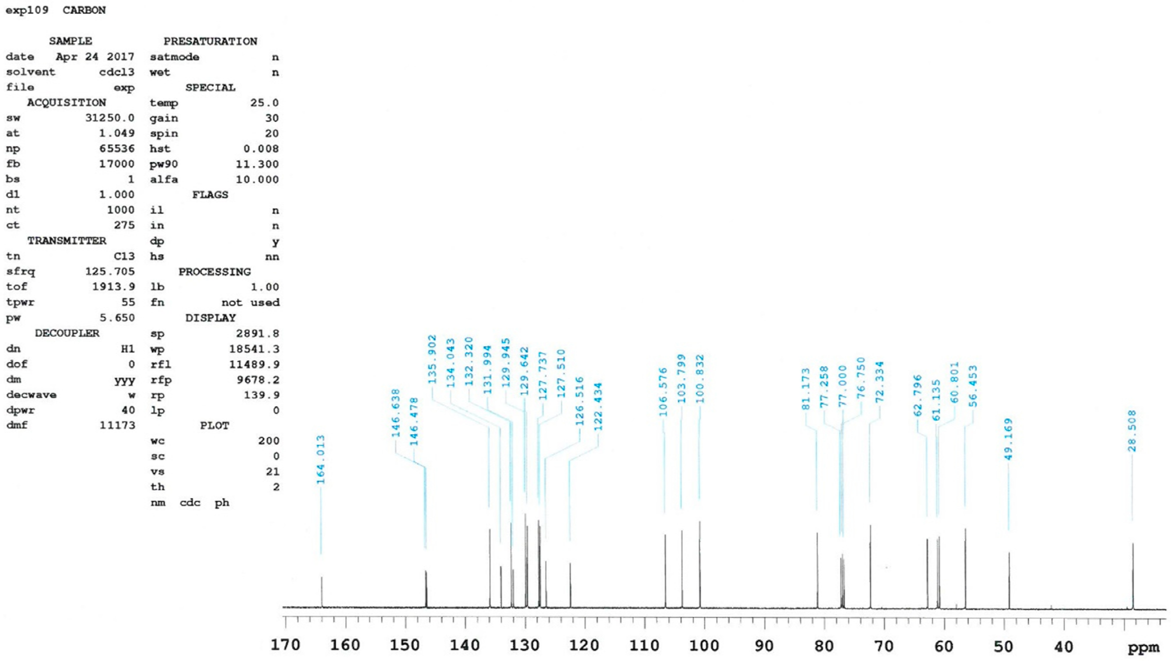

11-O-(2-methylbenzoyl)haemanthamine (1j)

ESI-HRMS

420.1733

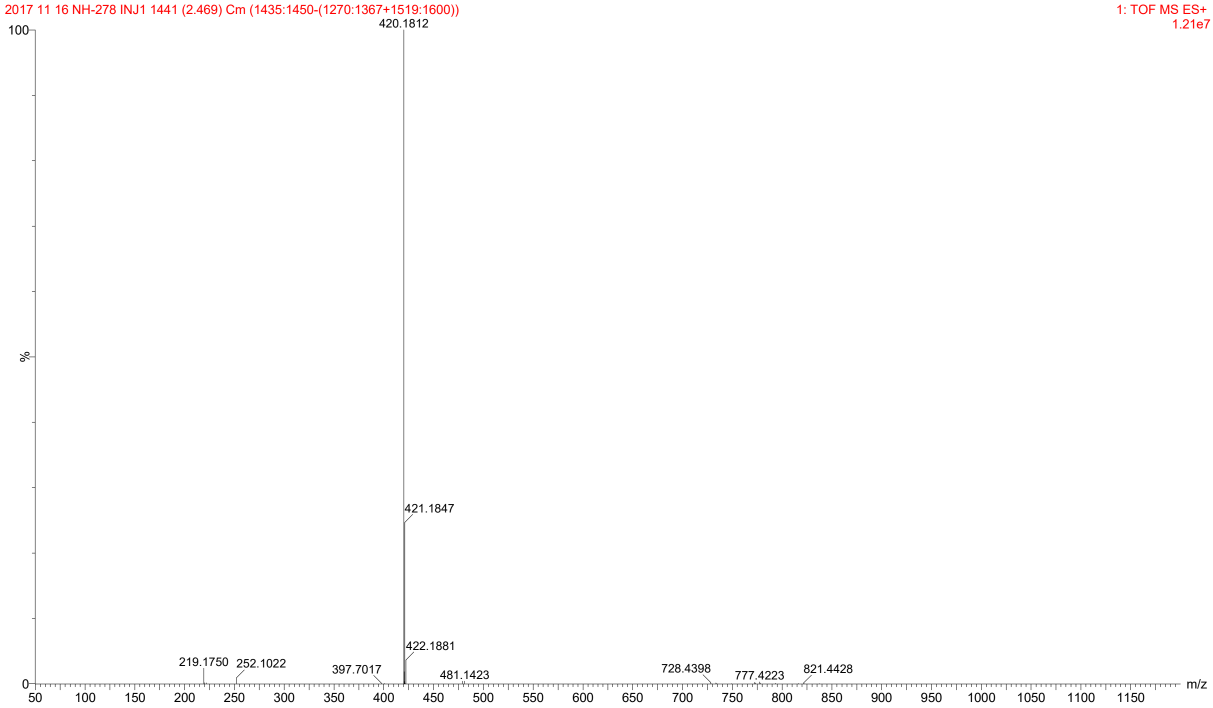

EI-MS

File :C:\msdchem\1\data\SNAP\3639LC.D
Operator :
Acquired : 17 Oct 2017 9:58 using AcqMethod LUCKA_7.M
Instrument : GCMS
Sample Name: LC-90
Misc Info :
Vial Number: 9

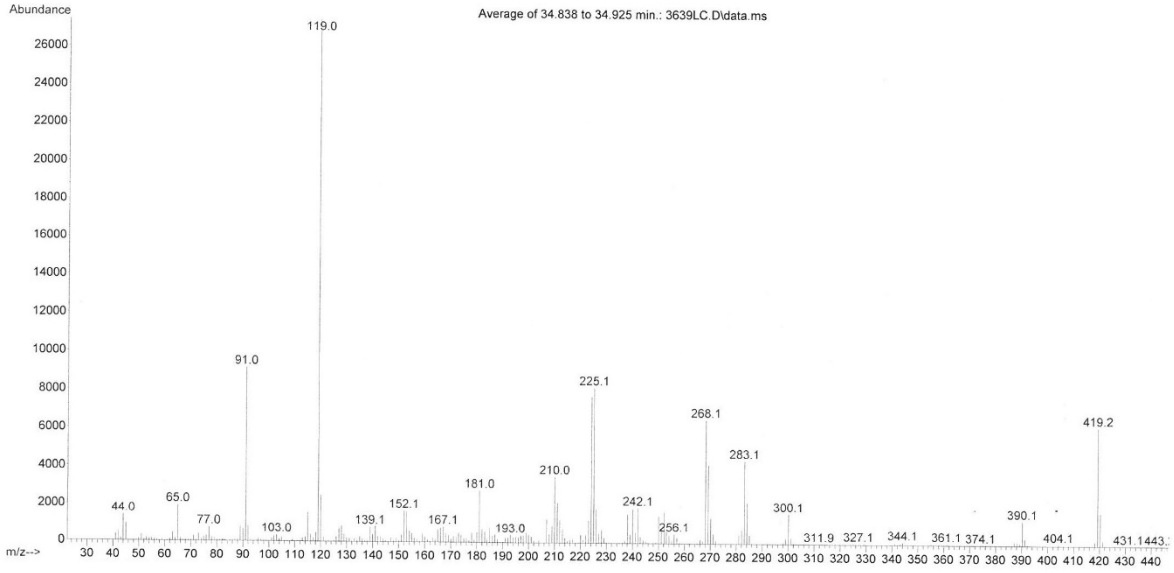

^{1}H-NMR

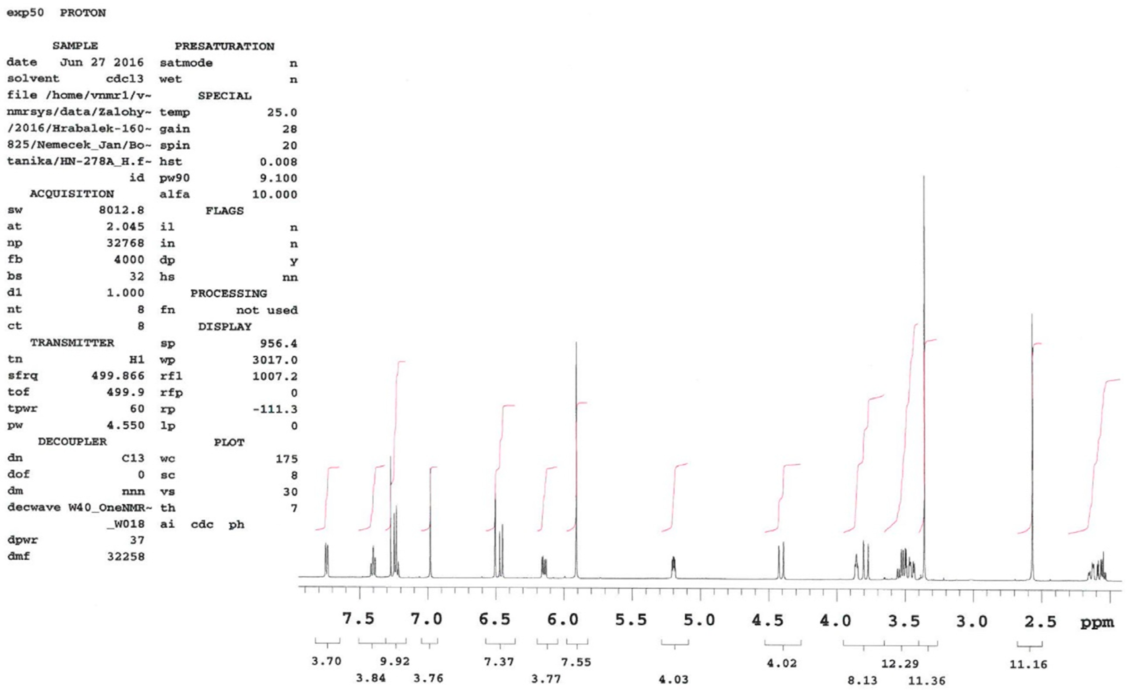

^{13}C-NMR

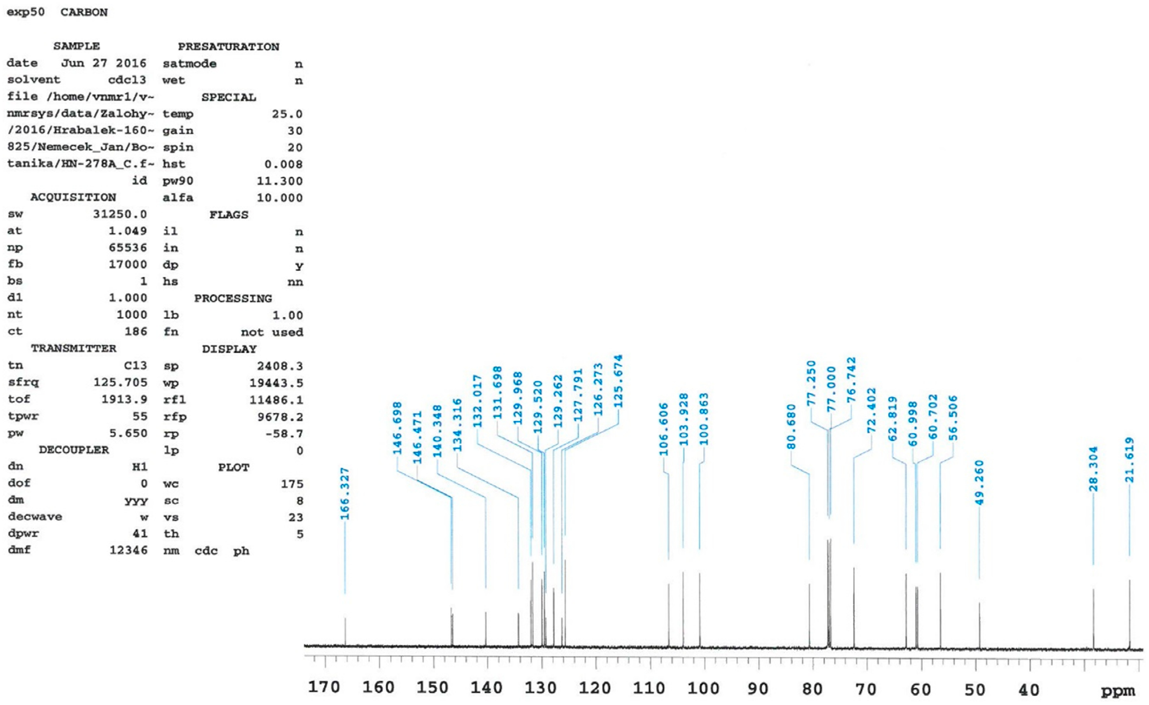

11-O-(3-methoxybenzoyl)haemanthamine (1k)

ESI-HRMS

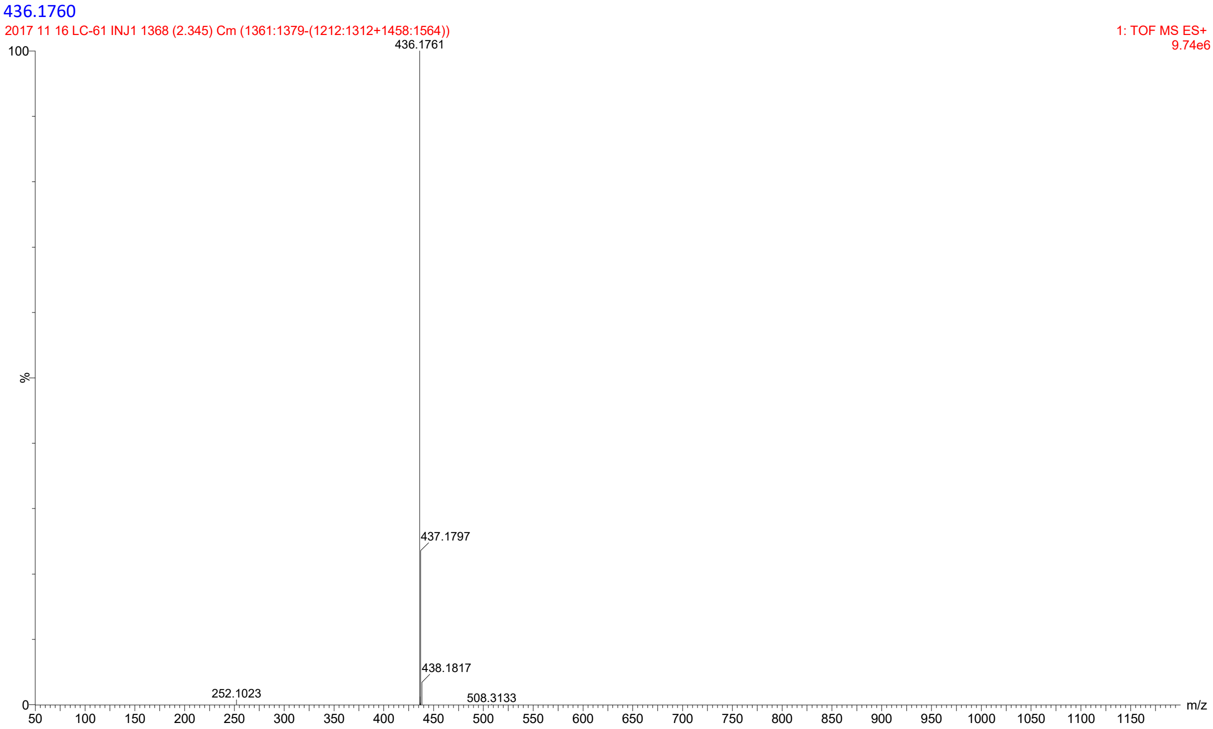

EI-MS

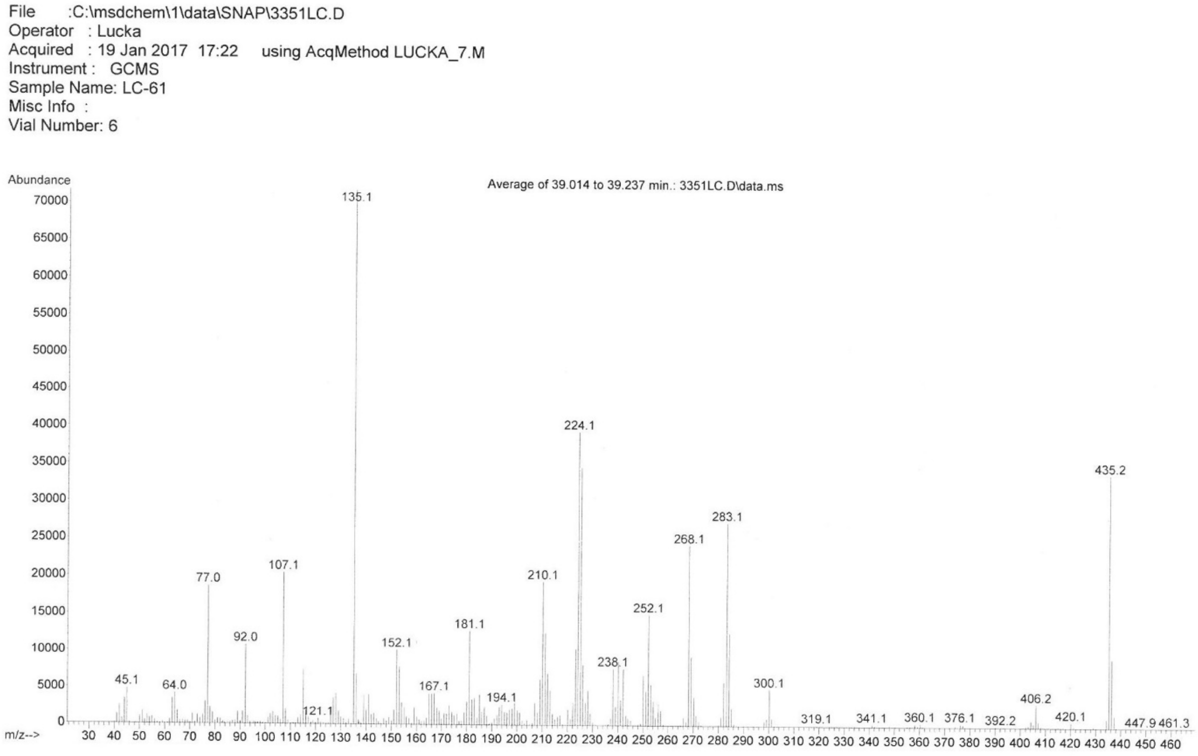

^{1}H-NMR

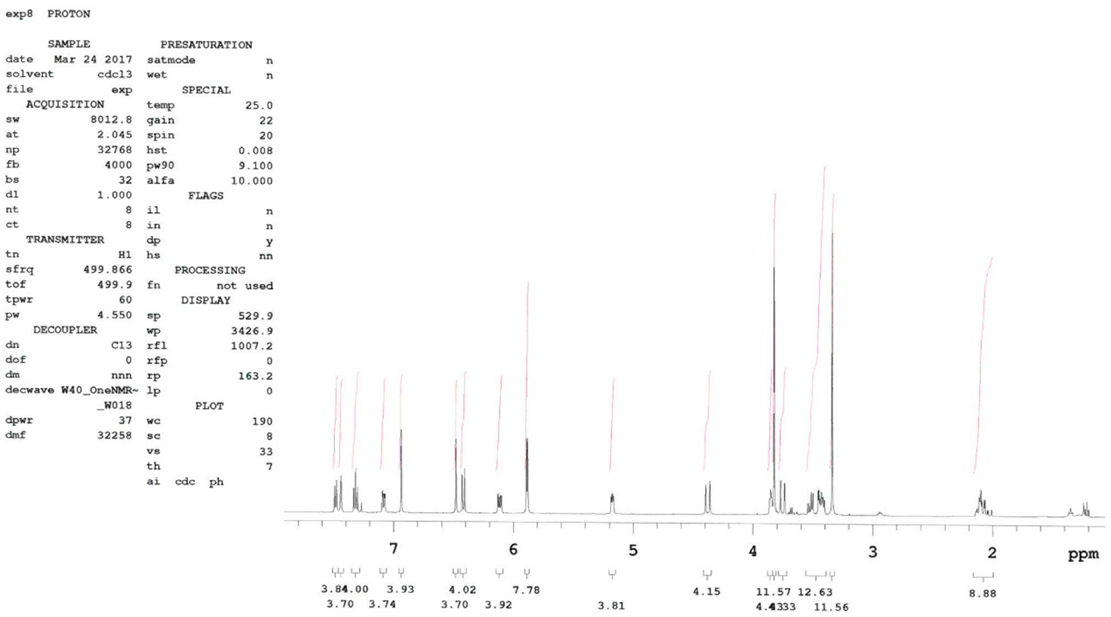

^{13}C-NMR

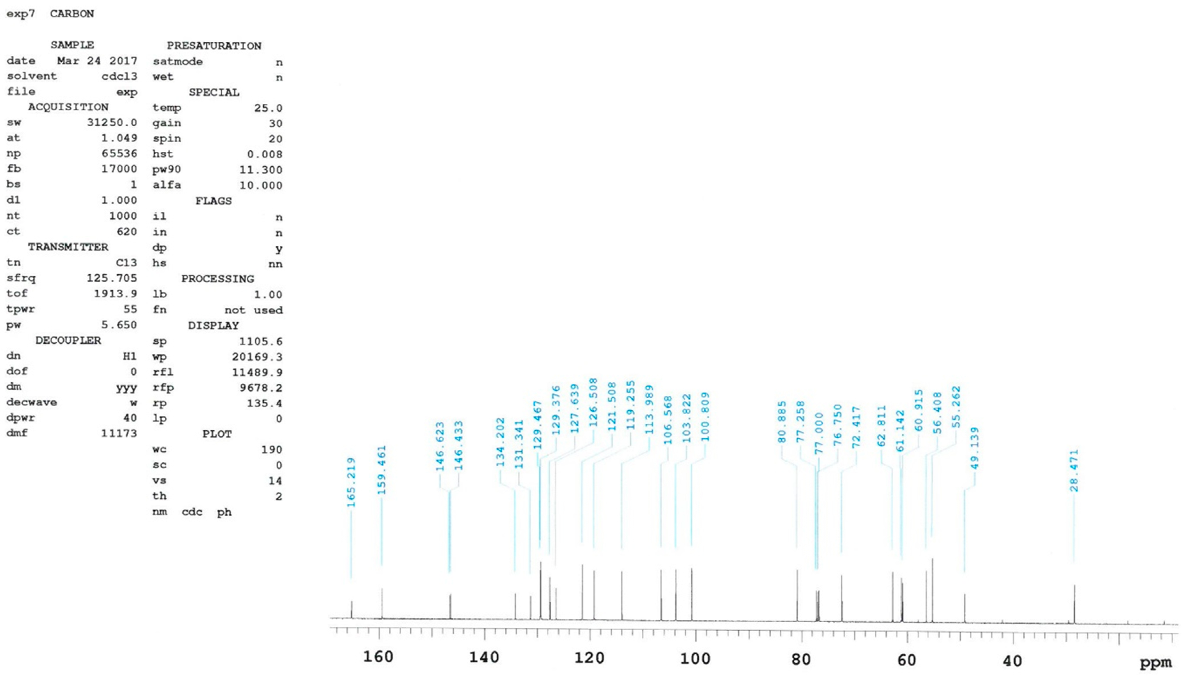

11-O-(4-nitrobenzoyl)haemanthamine (1m)

ESI-HRMS

451.1505

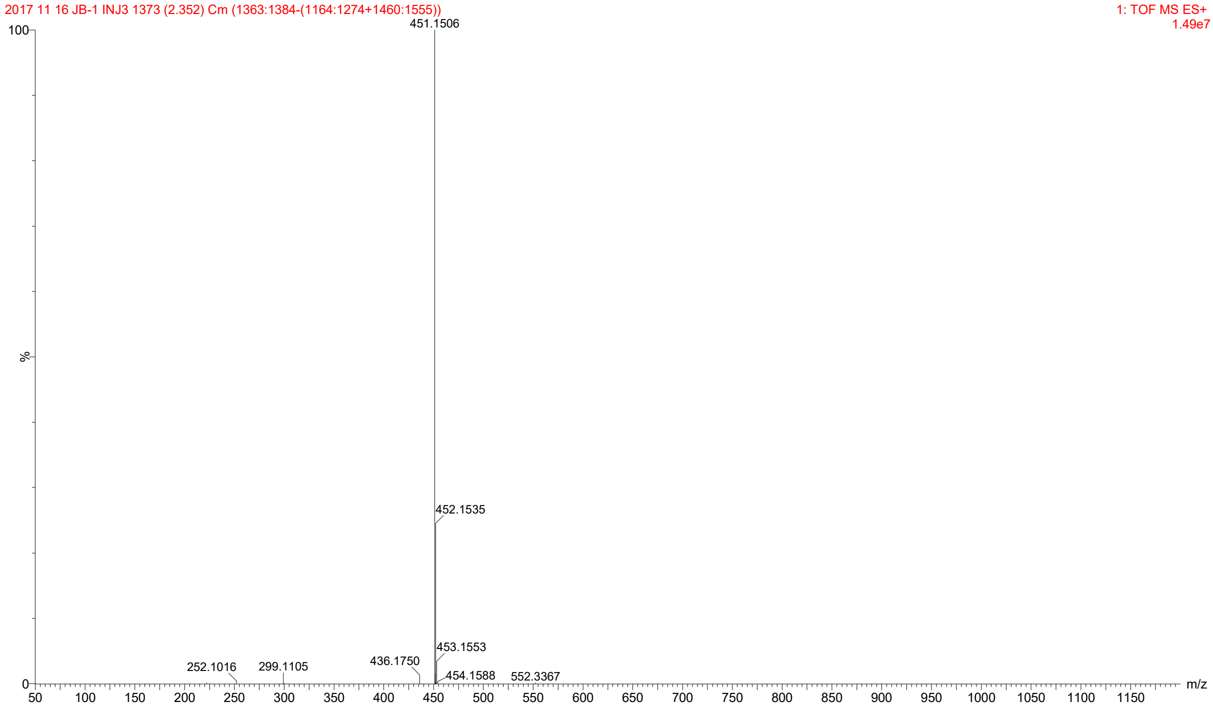

EI-MS

No ionization under EI-MS conditions

^{1}H-NMR

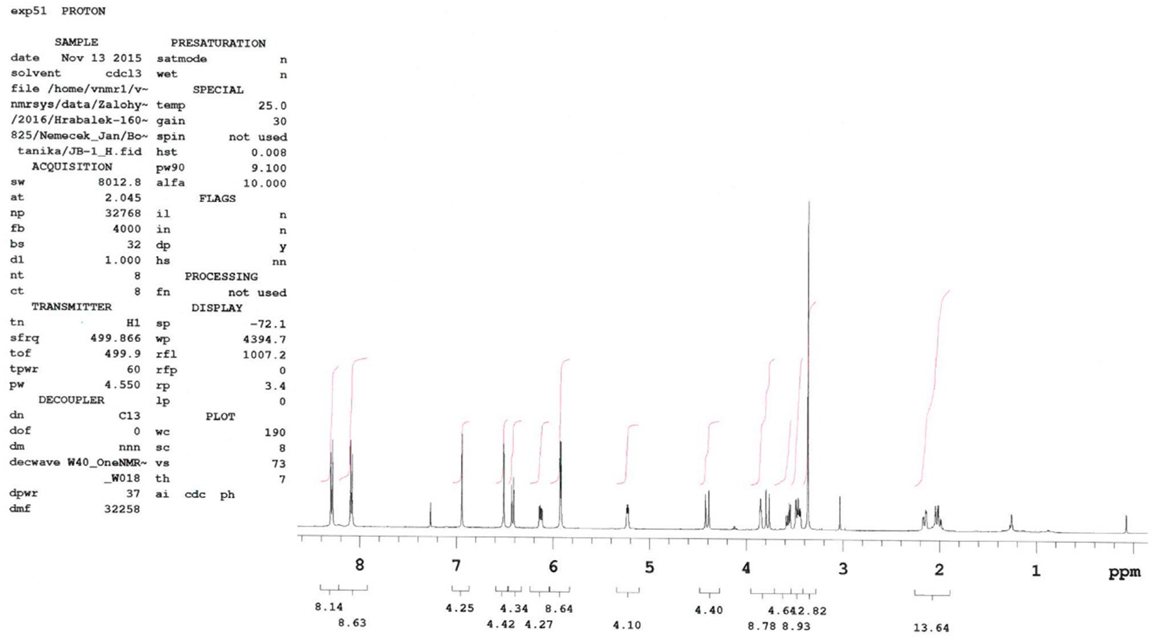

^{13}C-NMR

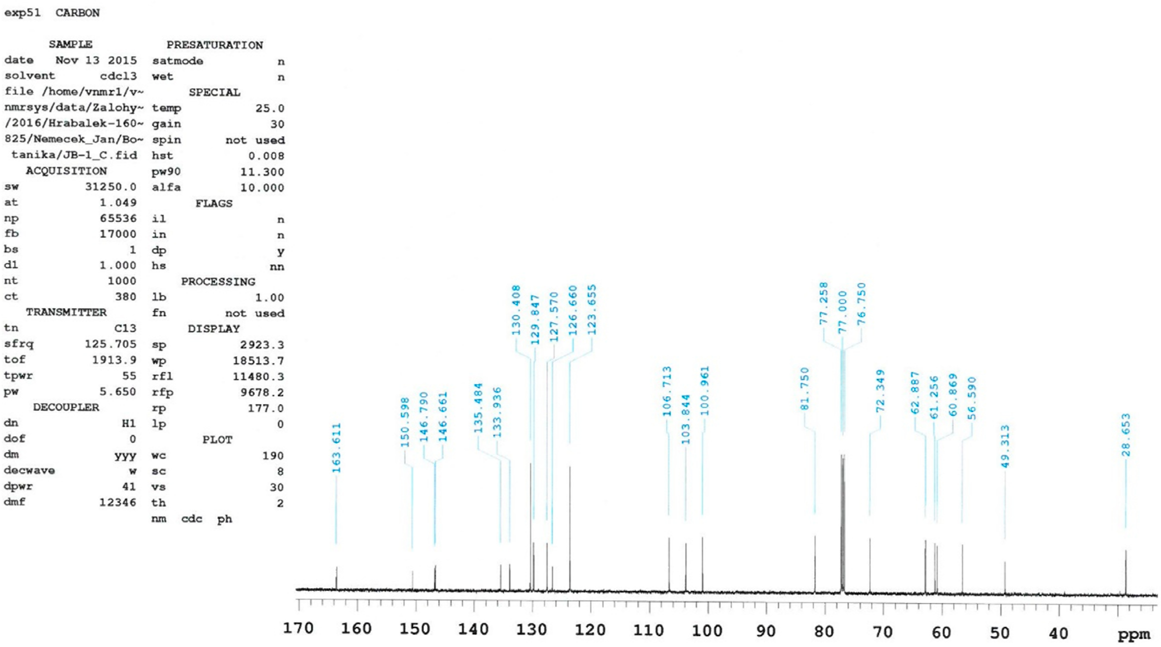
exp51 CARBON
SAMPLE
date Nov 13 2015
solvent cdcl3
file /home/vnmr1/v~
nmrsys/data/Zalohy~
/2016/Hrabalek-160~
825/Nemecek_Jan/Bo~
tanika/JB-1_C.fid
ACQUISITION
sw 31250.0
at 1.049
np 65536
fb 17000
bs 1
d1 1.000
nt 1000
ct 380
TRANSMITTER
tn C13
sfrq 125.705
tof 1913.9
tpwr 55
pw 5.650
DECOUPLER
dn H1
dof 0
dm yyy
decwave w
dpwr 41
dmf 12346
PRESATURATION
satmode n
wet n
SPECIAL
temp 25.0
gain 30
spin not used
hst 0.008
pw90 11.300
alfa 10.000
FLAGS
il n
in n
dp y
hs nn
PROCESSING
lb 1.00
fn not used
DISPLAY
sp 2923.3
wp 18513.7
rfl 11480.3
rfp 9678.2
rp 177.0
lp 0
PLOT
wc 190
sc 8
vs 30
th 2
nm cdc ph
163.611
150.598
146.790
146.661
135.484
133.936
130.408
129.847
127.570
126.660
123.655
106.713
103.844
100.961
81.750
77.258
77.000
76.750
72.349
62.887
61.256
60.869
56.590
49.313
28.653
170 160 150 140 130 120 110 100 90 80 70 60 50 40 ppm

Table S1. Permeability (*Pe* 10^{-6} cm s^{-1}) in the PAMPA-BBB assay for 10 commercial drugs (used in the experiment validation) and compounds **1g**, **1j** and **1m** with their predictive penetration in the CNS.[a]

Compound	Bibl.[b]	*Pe* (10^{-6} cm s^{-1})[c]	Prediction
Atenolol	0,8	0,3 ± 0,3	
Caffeine	1,3	0,1 ± 0,1	
Desipramine	12	7,7 ± 0,4	
Enoxacin	0,9	0,6 ± 0,5	
Hydrocortisone	1,9	0,8 ± 0,2	
Ofloxacin	0,8	0,3 ± 0,1	
Piroxicam	2,5	0,9 ± 0,2	
Promazine	8,8	8,6 ± 0,5	
Testosterone	17	13,6 ± 0,3	
Verapamil	16	15,5 ± 0,8	
**18g**		8,4 ± 0,2	CNS +
**18j**		5,8 ± 0,9	CNS +
**18m**		7,1 ± 0,3	CNS +

[a]PBS:EtOH (70:30) was used as solvent. [b]Reference Di et al. [c]Data are the mean ± SD of 2 independent experiments.

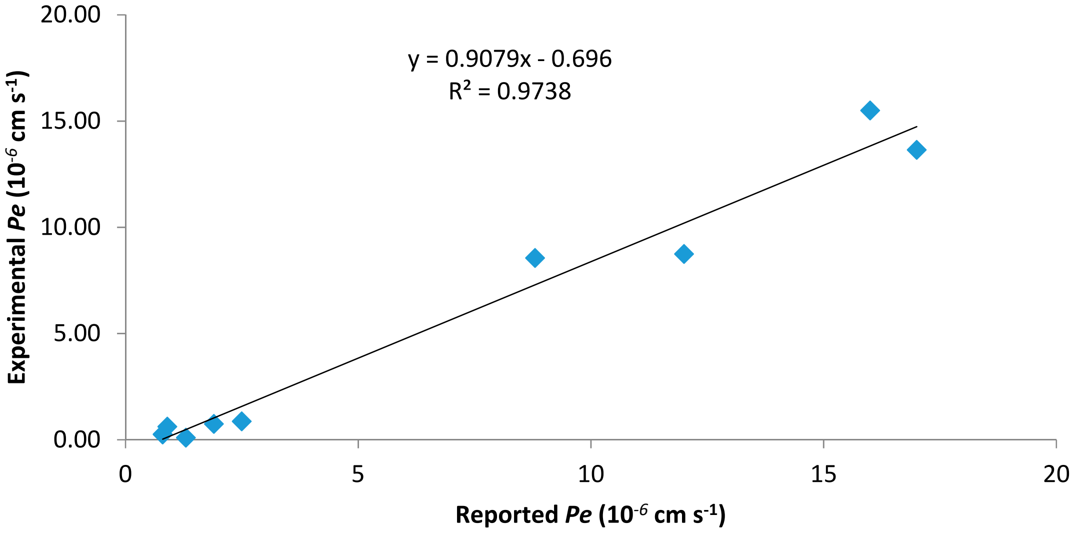

Figure S1. Linear correlation between experimental and reported permeability of commercial drugs using the PAMPA-BBB assay.

Table S2. Cell proliferation of haemanthamine derivatives (**1a – 1m;** c = 10μM) for 10 human cell lines.

	**1**	**1a**	**1b**	**1c**	**1d**	**1e**	**1f**	**1g**	**1**	**1i**	**1j**	**1k**	**1m**	**DOX**
**Jurkat**	13±3	100±13	104±3	110±3	92±1	100±7	90±12	83±11	69±6	133±10	95±11	83±1	69±7	0±3
**MOLT-4**	1±1	106±9	97±8	99±8	96±1	97±3	101±18	110±19	135±22	115±15	91±3	94±18	76±12	0±1
**A549**	30±6	95±9	84±3	84±3	99±7	105±9	106±12	98±2	75±25	122±5	96±8	102±5	74±6	66±16
**HT-29**	37±6	97±10	79±15	82±17	94±4	100±3	101±3	110±17	97±23	120±4	98±6	93±3	73±2	77±12
**PANC-1**	37±3	114±4	70±13	85±13	88±3	96±4	99±6	100±10	118±9	108±2	95±6	88±5	71±7	59±9
**A2780**	30±5	107±17	104±3	123±9	99±2	106±5	104±8	120±10	122±24	162±9	129±11	108±7	86±4	5±1
**HeLa**	16±2	83±6	69±11	74±8	93±6	98±3	88±3	98±2	72±4	93±5	104±4	84±12	71±1	7±10
**MCF-7**	11±1	97±19	85±7	103±7	93±5	95±4	96±13	108±10	90±11	105±4	112±3	100±2	62±10	41±7
**SAOS-2**	28±3	88±6	96±16	85±15	88±6	96±8	104±7	104±9	135±6	97±10	97±17	89±15	69±4	73±8
**MRC-5**	30±2	113±12	102±13	95±7	89±3	96±4	87±14	101±5	100±5	105±3	112±9	92±1	61±8	40±4

Values represent cell proliferation after haemanthamine analogue treatment and are expressed as percent of proliferation of untreated control cells. Each value is a mean of three independent experiments. Doxorubicin was used as a reference drug.
